# Genome-wide association and epistasis studies reveal the genetic basis of saline-alkali tolerance at the germination stage in rice

**DOI:** 10.3389/fpls.2023.1170641

**Published:** 2023-05-11

**Authors:** Guogen Zhang, Zhiyuan Bi, Jing Jiang, Jingbing Lu, Keyang Li, Di Bai, Xinchen Wang, Xueyu Zhao, Min Li, Xiuqin Zhao, Wensheng Wang, Jianlong Xu, Zhikang Li, Fan Zhang, Yingyao Shi

**Affiliations:** ^1^ College of Agronomy, Anhui Agricultural University, Hefei, Anhui, China; ^2^ Institute of Crop Sciences, Chinese Academy of Agricultural Sciences, Beijing, China; ^3^ National Nanfan Research Institute (Sanya), Chinese Academy of Agricultural Sciences, Sanya, Hainan, China

**Keywords:** germination stage, genome-wide association study, epistasis, rice, saline-alkali tolerance

## Abstract

**Introduction:**

Saline-alkali stress is one of the main abiotic factors limiting rice production worldwide. With the widespread use of rice direct seeding technology, it has become increasingly important to improve rice saline-alkali tolerance at the germination stage.

**Methods:**

To understand the genetic basis of saline-alkali tolerance and facilitate breeding efforts for developing saline-alkali tolerant rice varieties, the genetic basis of rice saline-alkali tolerance was dissected by phenotyping seven germination-related traits of 736 diverse rice accessions under the saline-alkali stress and control conditions using genome-wide association and epistasis analysis (GWAES).

**Results:**

Totally, 165 main-effect quantitative trait nucleotides (QTNs) and 124 additional epistatic QTNs were identified as significantly associated with saline-alkali tolerance, which explained a significant portion of the total phenotypic variation of the saline-alkali tolerance traits in the 736 rice accessions. Most of these QTNs were located in genomic regions either harboring saline-alkali tolerance QTNs or known genes for saline-alkali tolerance reported previously. Epistasis as an important genetic basis of rice saline-alkali tolerance was validated by genomic best linear unbiased prediction in which inclusion of both main-effect and epistatic QTNs showed a consistently better prediction accuracy than either main-effect or epistatic QTNs alone. Candidate genes for two pairs of important epistatic QTNs were suggested based on combined evidence from the high-resolution mapping plus their reported molecular functions. The first pair included a glycosyltransferase gene *LOC_Os02g51900 (UGT85E1)* and an E3 ligase gene *LOC_Os04g01490 (OsSIRP4)*, while the second pair comprised an ethylene-responsive transcriptional factor, *AP59 (LOC_Os02g43790)*, and a Bcl-2-associated athanogene gene, *OsBAG1 (LOC_Os09g35630)* for salt tolerance. Detailed haplotype analyses at both gene promoter and CDS regions of these candidate genes for important QTNs identified favorable haplotype combinations with large effects on saline-alkali tolerance, which can be used to improve rice saline-alkali tolerance by selective introgression.

**Discussion:**

Our findings provided saline-alkali tolerant germplasm resources and valuable genetic information to be used in future functional genomic and breeding efforts of rice saline-alkali tolerance at the germination stage.

## 1 Introduction

Soil saline-alkalization, a major factor limiting crop production, has become an increasingly serious global issue in recent years (Shabala, 2013; Wang et al., 2018a). Saline-alkalized soils have two coexisting abiotic stresses, salt stress and alkali stress. Salt stress is caused by neutral salts such as NaCl and Na_2_SO_4_, which can mainly cause Na^+^ toxicity, osmotic stress, and oxidative stress (Zhao et al., 2020). Alkali stress is primarily induced by alkaline salts such as NaHCO_3_ and Na_2_CO_3_ (Fang et al., 2021), resulting in an inhibition of plant growth through additional high pH stress, apart from the effects caused by salt stress (Zhang et al., 2017; Kaiwen et al., 2020). In fact, many reports indicate that alkali stress has more severe effects on crop plants than salt stress (Campbell and Nishio, 2000; Shi and Wang, 2005; Gao et al., 2008). Rice (*Oryza sativa* L.) is one of the most important food crops and is the primary food source for over half of the world’s population (Huang et al., 2013). Rice is sensitive to saline-alkali stress at different growth stages (Munns and Tester, 2008; Rao et al., 2013). Saline-alkali stress severely disrupts the growth and development of rice plants, resulting in reduced yields and thus is a major threat to world food security (Zhang et al., 2009). Globally, at least 900 million ha of abandoned land are otherwise suitable for crop production because of the severe saline-alkalized soils (Li et al., 2014). In the northeastern and northwestern China, rice growing areas have been expanding rapidly as the global warming. However, rice crops in these areas are facing increasingly more severe saline-alkali problem because of the fast evaporation of irrigated water of rice fields, in addition to ~9 million ha of heavily saline-alkalized wild lands that would have been used for rice production if the saline-alkali problem is overcome. Meanwhile, very limited breeding efforts have been taken to develop and adopt rice varieties tolerant to saline-alkali stress, due partially to “unavailability” of rice germplasm accessions highly tolerant to saline-alkali stresses, and partially to our poor understanding the genetic basis of rice tolerance to saline-alkali stresses.

Involving complex physiological and molecular mechanisms (Ganapati et al., 2022), rice saline- alkali tolerance is expected to be controlled by many genes or quantitative trait loci (QTLs). To facilitate breeding efforts for developing saline-alkali tolerant rice varieties for expanding rice growing areas. There are increasing interests to identify the genes and QTLs responsible for saline-alkali tolerance in rice. However, past efforts have been primarily focused on genetic and molecular dissection of salt tolerance (ST) in rice. To date, at least 85 QTLs and genes related to rice salt tolerance have been identified by conventional QTL mapping and map-based cloning from mutants (Yao et al., 2005; Sabouri and Sabouri, 2008; Sabouri et al., 2009; Thomson et al., 2010; Wang et al., 2012; Quan et al., 2017; Dai et al., 2022). According to the funRiceGenes database (Huang et al., 2022), at least 383 genes are associated with salt tolerance, such as *SKC1* (Ren et al., 2005), *DST* (Huang et al., 2009), *HST1* (Takagi et al., 2015), *qSE3* (He et al., 2019). In contrast, few efforts have been made to understand the genetic basis of alkali tolerance in rice. Li et al. (2020) identified a major QTL, *qAT11* on chromosome 11, for alkali tolerance at the bud stage using a population of 184 recombinant inbred lines. Wang et al. (2019) reported that *OsPPa6*, an osmotic regulatory factor, positively regulates alkaline tolerance rice. Guo et al. (2014) demonstrated that *ALT1*, encoding the Snf2 family chromatin remodeling ATPase, negatively regulates alkali tolerance mainly by enhancing the defense system against oxidative damage in rice.

Genome-wide association studies (GWAS) overcome two main limitations of QTL mapping by traditional biparental genetic populations in plants: (i) providing relatively higher mapping resolution due to plenty of historical recombination events and (ii) using the natural population with more genetic diversity (Korte and Farlow, 2013). Recently, GWAS have become an effective and powerful tool for identifying genes associated with complex traits in rice (Huang et al., 2010; Shi et al., 2017; Liu and Yan, 2019; Zhang et al., 2019; Liu et al., 2021b). In the past decade, GWAS has been successfully used to detect the potential loci for salt tolerance at various developmental stages in rice, which has been well summarized by a recent review (Dai et al., 2022). In contrast, there are few studies on the potential loci for alkali tolerance. Mei et al. (2022) identified 90 loci associated with alkali tolerance at the germination stage by GWAS using 428 diverse rice accessions. Li et al. (2019) detected eight QTLs significantly associated with alkali tolerance-related traits at the seedling stage by GWAS using 295 *Geng* (*japonica*) varieties.

Although some main-effect loci for saline-alkali tolerance have been identified via GWAS in rice, the genetic architecture of rice saline-alkali tolerance remains unclear since genome-wide epistatic interactions (i.e., QTN by QTN interactions [QQIs]) have been ignored in most GWAS. Epistasis is pervasive and has been recognized as a key aspect of plant genetic architecture (Brachi et al., 2011; Korte and Farlow, 2013; Doust et al., 2014; Mackay, 2014). Recent studies have demonstrated that identifying epistatic interactions in diverse plant germplasm resources can retrieve a large proportion of the “missing heritability”, thereby improving phenotypic prediction accuracy and providing a comprehensive genetic basis for complex traits (Maurer et al., 2015; Luo et al., 2017; Carré et al., 2022). With the rapid population size and marker density increase, GWAS faces statistical and computational challenges in detecting robust epistatic interactions. Fortunately, several new statistical methods and software packages have been developed to identify epistatic interactions in GWAS with high computational efficiency and low false positive rate. Recently, Li et al., 2022a; Li et al., 2022b proposed a powerful and accurate approach, IIIVmrMLM, by integrating the compressed variance component mixed model with the multi-locus random-SNP-effect mixed linear model for detecting QTNs and QQIs in GWAS.

To unravel the complex genetic mechanisms underlying saline-alkali tolerance in rice, we conduct a GWAS by the IIIVmrMLM method to detect the genome-wide main-effect QTNs and QQIs associated with seven saline-alkali tolerance-related traits at the germination stage using a large panel of 736 diverse rice accessions from the 3,000 rice genome project (3KRG). By identifying large numbers of QTNs and their QQIs, our findings greatly enriched the current knowledge on the genetic architecture of saline-alkali tolerance in rice and provided valuable germplasm resources for developing saline-alkali tolerant varieties through molecular breeding.

## 2 Materials and methods

### 2.1 Plant materials

A total of 736 accessions from the 3,000 rice genome project (3KRG) were used to evaluate saline-alkali tolerance of rice at the germination stage. Based on the known population structure (Wang et al., 2018b), these accessions belong to 12 subgroups, including *admix* (*adm*) (4 accessions), *Aus* (160 accessions), *Basmati* (67 accessions), *Geng/japonica* (*GJ*)*-adm* (8 accessions), *GJ-sbtrp* (65 accessions), *GJ-tmp* (58 accessions), *GJ-trp* (50 accessions), *Xian/indica* (*XI*)*-adm* (78 accessions), *XI-1A* (56 accessions), *XI-1B* (59 accessions), *XI-2* (61 accessions) and *XI-3* (70 accessions) (**Supplementary Table 1**).

### 2.2 Phenotypic evaluation of saline-alkali tolerance at the germination stage

To evaluate saline-alkali tolerance of the rice accessions at the germination stage, we measured seven germination-related traits following the method of Mei et al. (2022). Briefly, surface sterilized seeds that had been broken dormancy were treated with 0.15% Na_2_CO_3_ solution (pH = 11) as the saline-alkali stress treatment at the germination stage and with distilled water as the control. Three biological replicates for each accession were performed. In each replicate, 20 seeds of each accession were evenly placed in a 90-mm diameter Petri dish with filter paper, then 10 mL of 0.15% Na_2_CO_3_ solution or distilled water was added. We placed the Petri dishes in a growth chamber under the 14-h light/10-h dark photoperiod (28°C/26°C) with 70% relative humidity conditions for 7 d and then changed the Na_2_CO_3_ solution and distilled water daily.

Germination of seeds was considered to have taken place when the root length was equal to the grain length and the shoot length was equal to half the grain length. We counted germinated seeds under the saline-alkali stress and control conditions every day. The germination potential (GP) and germination rate (GR) of each accession in each replicate were calculated as the proportion of germinated seeds (out of the 20 seeds originally sown) on day 3 and day 7, respectively. Then, germination index (GI) was calculated as GI = Σ(G_t_/T_t_), and mean germination time (MGT) was calculated as MGT = ΣT_t_N_t_/ΣN_t_, where G_t_ is the accumulated number of the germinated seeds on day t, T_t_ was the time corresponding to G_t_ in days, and N_t_ was the number of newly germinated seeds on day t (Alvarado et al., 1987; Wang et al., 2010). On day 7, shoot length (SL) and root length (RL) of eight seeds from each accession per replicate in the same growth trend were measured with a ruler. We calculated vigor index (VI) as follows: VI = (mean SL) × GI. The ratios of the saline-alkali stress to the control conditions for the seven germination-related traits, namely relative mean germination time (RMGT), relative germination index (RGI), relative germination potential (RGP), relative germination rate (RGR), relative root length (RRL), relative shoot length (RSL), and relative vigor index (RVI) were used to evaluate rice saline-alkali tolerance during germination.

### 2.3 Identification of main-effect QTNs

The 3KRG 1M GWAS SNP dataset was downloaded from the Rice SNP-Seek Database (Alexandrov et al., 2015). We used PLINK 1.9 (Purcell et al., 2007) to obtain a subset of 610,943 SNPs with a missing ratio < 20% and minor allele frequency > 5% for GWAS. The “Single_env” method of IIIVmrMLM (Li et al., 2022a) was used to identify main-effect QTNs associated with saline-alkali tolerance based on a multi-locus GWAS model. The parameters of IIIVmrMLM function were set to “SearchRadius = 20, svpal = 0.01 and num_Threads = 8”. Principal component analysis was performed by GCTA (Yang et al., 2011) with the “-make-grm” parameter and the first five principal components were used to control population structure. The significance thresholds of association by the Bonferroni correction method (0.05/*m*) were calculated for claiming significant QTNs for the whole panel (*P* = 8.18 ×10^-8^), where *m* is the number of SNPs. The SNP with the minimum *P* value at a locus was considered the lead SNP. Based on reported genome-wide linkage disequilibrium (LD) decay in 3KRG (Wang et al., 2018b), the other significant SNPs within a range of 150 kb on each side of a lead SNP were merged as a single association locus. The local LD block analysis was performed within 200 kb upstream and downstream of the lead SNP by the “–blocks” function with the default parameter in PLINK 1.9 (Purcell et al., 2007). The heatmap of pairwise LDs was plotted using the LDBlockShow (Dong et al., 2021).

### 2.4 Identification of epistatic QTNs

To identify QTN by QTN interactions (QQIs) associated with saline-alkali tolerance-related traits during rice germination, we selected the SNPs with *P* < 0.01 for each trait except RMGT based on the above multi-locus GWAS. For computational reasons, we screened the SNPs with *P* < 1×10^-5^ for RMGT. Then, the “Epistasis” method of IIIVmrMLM was used to detect epistatic QTNs. The parameters of the IIIVmrMLM function were set to “SearchRadius = [0, 1], svpal = [0.10, 0.10] and sblgwas_t = -2.50” and num_Threads = 8”. Principal component analysis was performed by GCTA (Yang et al., 2011) with the “-make-grm” parameter and the first five principal components were used to control population structure. The threshold for significant epistatic QTNs was set at LOD = 3.0. The QQIs results were plotted into circos diagrams using the online website https://www.omicstudio.cn/tool/50 (Gu et al., 2014).

### 2.5 Candidate gene prediction

Candidate genes in a QTN locus associated with saline-alkali tolerance were determined based on the following criteria: (1) excluding genes encoding transposon, retrotransposon and hypothetical proteins based on the annotation of Nipponbare reference genome IRGSP 1.0 (Kawahara et al., 2013); (2) significantly regulated (gene expression fold-change > 4.0) by the saline-alkali stress according to Plant Public RNA-seq Database (Yu et al., 2022); and (3) significant phenotypic differences between different gene-haplotypes, which consisted of all SNPs within its 1 kb upstream region and the coding sequence region (ignored synonymous SNPs) (Zhang et al., 2021). Only major haplotypes carried by at least 15 rice accessions were used for phenotypic multiple comparisons by Duncan’s multiple range *post-hoc* tests with the “agricolae” package in R.

### 2.6 RNA extraction and qRT-PCR analysis

To detect expression levels of the candidate genes of some main-effect and epistatic QTNs, we selected two saline-alkali sensitive accessions (American Huangkedao [*Geng*] and ARC 6052 [*Xian*]) and two saline-alkali tolerant accessions (SACHIKAZE [*Geng*] and ARC 18597 [*Xian*]) from the GWAS panel. After 24 h of saline-alkali stress with 0.15% Na_2_CO_3_, seed embryos of each accession were sampled under saline-alkali stress and control conditions, respectively. Three biological replicates for each accession were performed. Total RNA was extracted from seed embryos using plant RNA extraction kit (Tiangen Biotechnology), and reverse transcribed using reverse transcription kit (Tiangen Biotechnology). Real-time qRT-PCR analyses were conducted with Taq Pro Universal SYBR qPCR Master Mix (Vazyme, Q712-02). *UBQ* was used as the internal control and the relative expression levels of the target genes were calculated using the 2^-ΔΔCT^ method (Livak and Schmittgen, 2001). All primers used for qRT-PCR are listed in **Supplementary Table 2**.

### 2.7 Datasets used for comparison of genomic prediction

To determine the impact of epistatic QTNs on genomic prediction, we compared the prediction abilities based on different QTN datasets for each trait using the R package “rrBLUP” with the “mixed.solve” function (Endelman, 2011). The prediction abilities of the main-effect QTNs, QQIs and their combinations for each trait were obtained based on 10 times repeated 10-fold cross-validation, respectively. The missing genotypes of QTNs were replaced by their mean values. Two-sided Wilcoxon rank-sum tests were used to test the differences among the prediction abilities.

### 2.8 Statistical analysis

One-way ANOVA was used to test the phenotypic differences for measured traits between subpopulations by the R package “agricolae”. Spearman’s correlation coefficients between different saline-alkali tolerance traits were calculated using the R package “ggcor”. Boxplots and barplots on the measured trait variation in different rice subpopulations were generated using the R “ggplot2” package. The haplotypic enrichment analysis in a subpopulation was performed using the function “phyper” in R.

## 3 Results

### 3.1 Phenotypic variations of saline-alkali tolerance in different rice subpopulations

**Figure 1:**
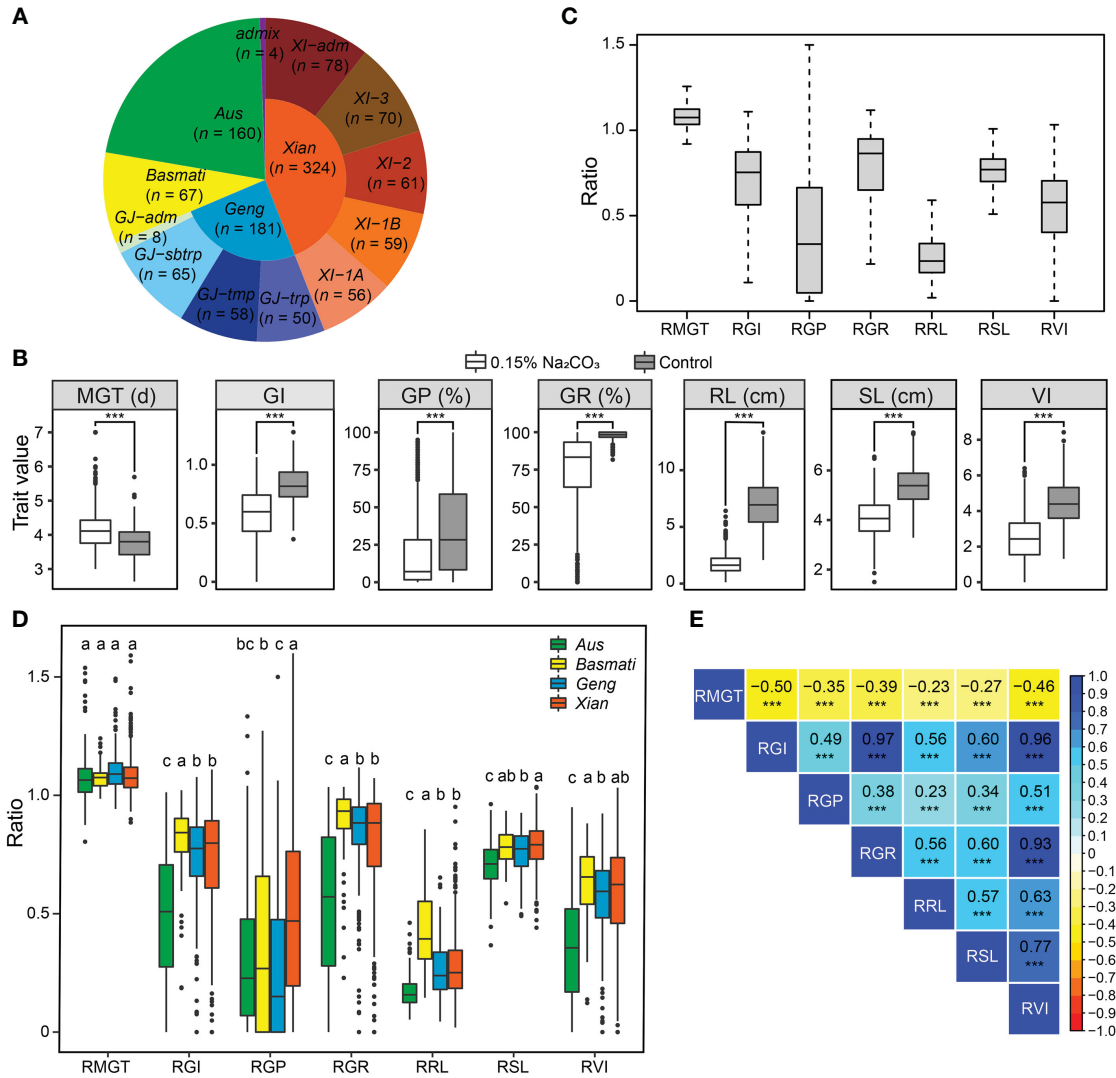
Phenotypic variations of saline-alkali tolerance-related traits at the germination stage. **(A)** The proportion of each subpopulation of the 736 rice accessions used in this study. **(B)** Phenotypic distribution of mean germination time (MGT), germination index (GI), germination potential (GP), germination rate (GR), root length (RL), shoot length (SL), and vigor index (VI) under saline-alkali stress and control conditions. ***P < 0.001 (two-tailed Student’s *t*-test). **(C)** Distribution of relative saline-alkali damage of the measured traits (stress/control) in the whole population. Relative mean germination time (RMGT), relative germination index (RGI), relative germination potential (RGP), relative germination rate (RGR), relative root length (RRL), relative shoot length (RSL), and relative vigor index (RVI). **(D)** Distribution of relative saline-alkali damage of the measured traits in the subpopulations *Geng*/*japonica*, *Xian*/*indica*, *Aus*, and *Basmati*. Different letters above the boxplot indicate significant differences according to Duncan’s multiple range *post-hoc* test (*P* < 0.05). **(E)** Correlations between the measured saline-alkali tolerance-related traits in the whole population, in which the numbers in the middle of the cells are the correlation coefficients and *** indicate significant correlations at *P* < 0.001.

To evaluate saline-alkali tolerance of the 736 diverse rice accessions at the germination stage (**Figure 1A**), we measured seven germination-related traits (MGT, GI, GP, GR, RL, SL, and VI) under control and saline-alkali stress conditions. As shown in **Figure 1B**, GI, GP, GR, RL, SL, and VI were significantly reduced and MGT was significantly prolonged under saline-alkali stress compared to the control, indicating that saline-alkali stress significantly inhibited rice germination and growth and the measured traits were indeed related to rice saline-alkali tolerance. The degree of saline-alkali damage to these germination-related traits was as follows: RL, GP, VI, GI, GR, SL, and MGT (**Figure 1C**). Of these, RL, the trait most affected by saline-alkali stress, was reduced by an average of 73.1% across the 736 accessions.

To eliminate the influence of genotype on the development of germination traits, we used the relative trait value (stress/control) to evaluate the saline-alkali tolerance of rice accessions. A wide range of variation in the saline-alkali tolerance traits was observed among different rice populations and among different accessions within each of the populations (**Figure 1D**). At the population level, the overall rank of saline-alkali tolerance was *Basmati* > *Xian* > *Geng* > *Aus*, which varied slightly depending on specific saline-alkali tolerance traits (**Figure 1D**). There was no significant difference in RMGT among the four subpopulations. For the other six traits, the *Basmati* accessions had the highest mean values in four traits except for RGP and RSL, and the *Aus* accessions had the lowest mean values in five traits except for RGP. The *Xian* accessions had significantly higher RGP and RSL than the *Geng* accessions (**Figure 1D**). Among the three *Geng* subgroups, *GJ-tmp* accessions had significantly higher RGP and RSL than the *GJ-sbtrp* and *GJ-trp* accessions, while the *GJ-trp* accessions had significantly higher RRL than the *GJ-sbtrp* and *GJ-tmp* accessions. Among the four *Xian* subgroups, the *Xian-3* subgroup had the strongest saline-alkali tolerance, while *Xian-1B* had the weakest saline-alkali tolerance (**Supplementary Figure 1**). These findings suggest that identifying loci associated with saline-alkali tolerance and their corresponding favorable alleles from diverse subpopulations is valuable in molecular breeding for rice saline-alkali tolerance. Of the 736 accessions evaluated, 30 accessions from ten countries showing high levels of saline-alkali tolerance at the germination stage were screened according to the two traits of RRL and RGP, including 7 *Basmati* accessions, 21 *Xian* accessions, 1 *Aus* accession and 1 *Geng* accession, respectively (**Supplementary Table 1**), providing a valuable source of materials for future theoretical and breeding research on saline-alkali tolerance of rice.

As expected, moderate negative correlation existed between the measured saline-alkali tolerance-related traits (RGR, RGI, RVI, RSL and RRL) and RMGT, while positive correlation existed between RGR, RGI, RVI, RSL and RRL, though much stronger between RGI, RGR and RVI (**Figure 1E**). Similar results were found in different subpopulations, though the trait correlation was stronger in population *Xian* than in other populations (**Supplementary Figure 2**).

### 3.2 Main-effect QTNs associated with saline-alkali tolerance at the germination stage

**Figure 2:**
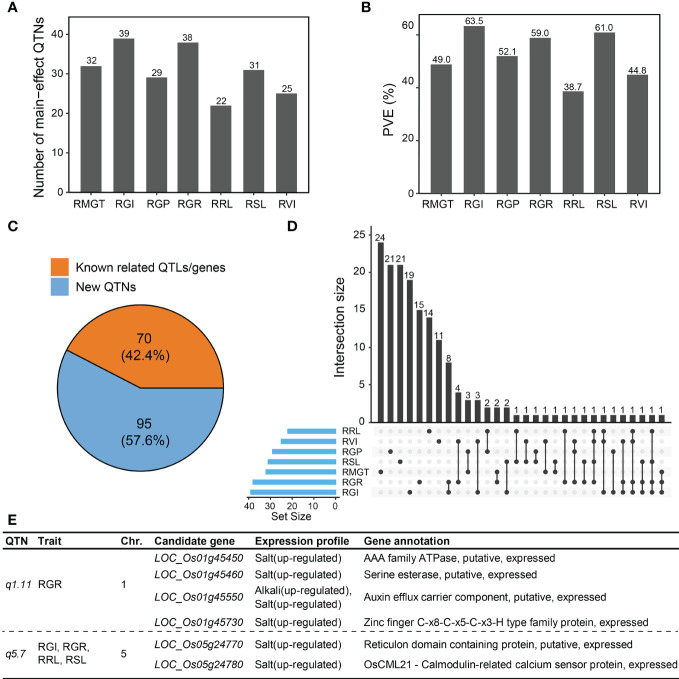
Main-effect QTNs associated with saline-alkali tolerance at the germination stage. The number of main-effect QTNs **(A)** and cumulative PVE **(B)** for different traits. **(C)** Different ratios for main-effect QTNs linked with known QTLs related to saline-alkali tolerance and genes related to saline-alkali tolerance in rice. Orange color, the number of known QTLs/genes in main-effect QTNs; blue color, the number of main-effect QTNs without any known QTLs/genes. **(D)** Intersection size of main-effect QTNs between different traits. **(E)** Candidate genes of two important main-effect QTNs, *q1.11* and *q5.7*.

Using the IIIVmrMLM software, 165 QTNs associated with saline-alkali tolerance-related traits at the germination stage were identified based on a multiple-locus GWAS (**Supplementary Table 3**). Of these, 32, 39, 29, 38, 22, 31 and 25 QTNs were detected for RMGT, RGI, RGP, RGR, RRL, RSL, and RVI, respectively, which explained 49.0%, 63.5%, 52.1%, 59.0%, 38.7%, 61.0% and 44.8% of total trait phenotypic variation, respectively (**Figures 2A, B** **)**. A total of 70 (42.4%) main-effect QTNs co-localized with previously reported QTLs related to alkali tolerance and genes related to saline-alkali tolerance in rice, suggesting that most QTNs detected by the multi-locus random-SNP-effect mixed linear model were reliable (**Figure 2C**). The large number of main-effect QTNs identified in this study also provided evidence for the complexity of the genetic mechanisms underlying saline-alkali tolerance during rice germination. Interestingly, the same QTNs were significantly associated with two or more saline-alkali tolerance-related traits (RGR, RGI, RVI, RSL and RRL) in 29 cases (**Figure 2D**). For example, a main-effect QTN on chromosome 5, *q5.7*, was commonly associated with RGR, RGI, RSL, and RRL, explaining a small portion of phenotypic variation (2.69% for RGR, 4.68% for RGI, 3.43% for RSL, and 2.59% for RRL). In addition, the most significant main-effect QTN associated with RGR, *q1.11*, explained 4.49% of the phenotypic variation of RGR.

### 3.3 Candidate genes of important main-effect QTNs *q1.11* and *q5.7*

**Figure 3:**
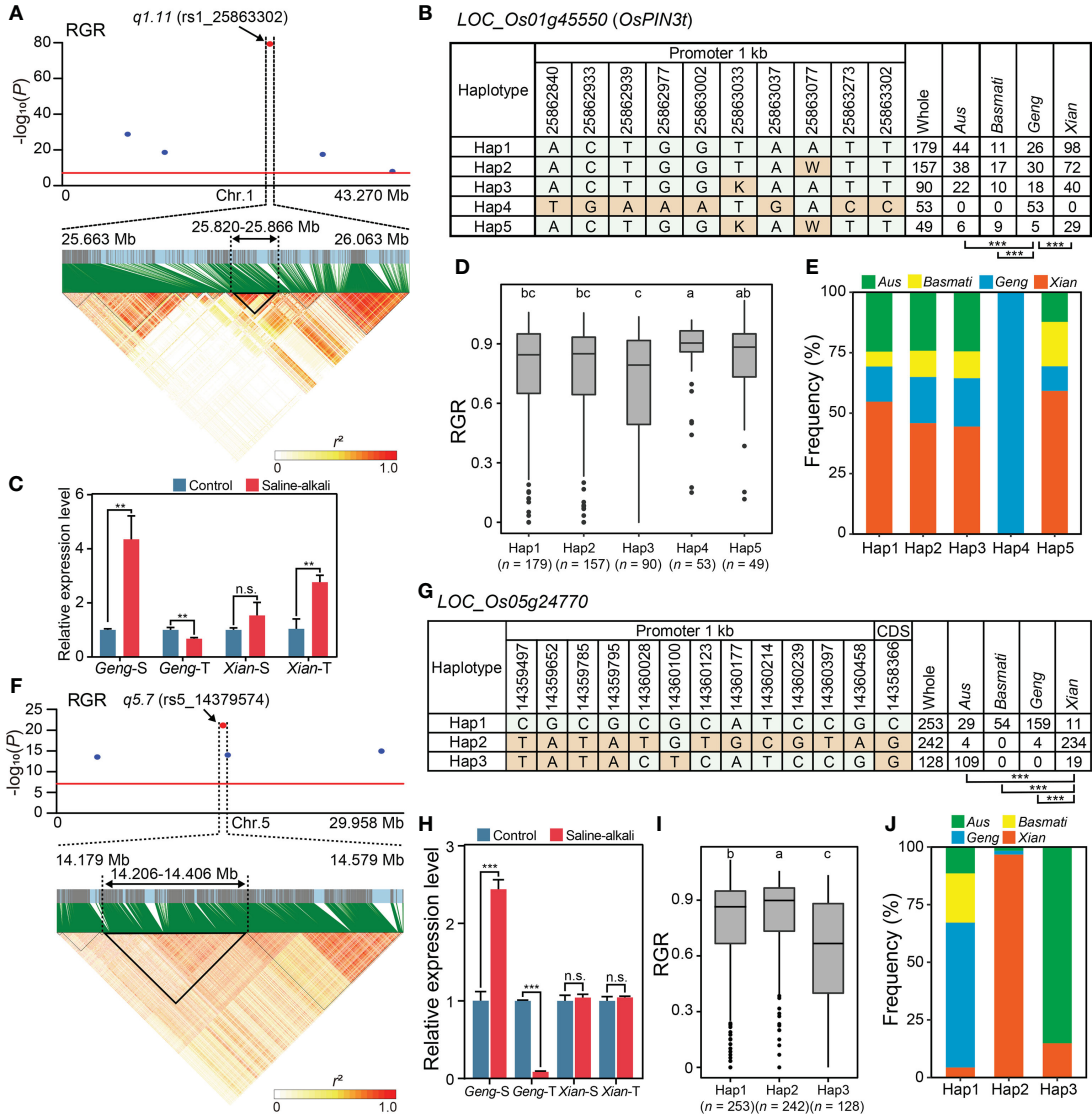
Promising candidate genes of *q1.11* on chromosome 1 and *q5.7* on chromosome 5. **(A)** Local Manhattan plot (top) for RGR and LD heat map (bottom) of *q1.11*. The red dot indicates the lead SNP rs1_25863302 and the position of the promising candidate gene *LOC_Os01g45550* (*OsPIN3t*). **(B)** The haplotypes in the 1 kb of the upstream promoter region of *LOC_Os01g4555*0. **(C)** The relative expression levels of *LOC_Os01g4555*0 under saline-alkali stress for 24 h and control conditions. Gene expression was normalized to that of the *UBQ* gene control. **(D)** The distribution of RGR for the five haplotypes of *LOC_Os01g45550*. **(E)** Frequency of five haplotypes of *LOC_Os01g45550* in subpopulations. **(F)** Local Manhattan plot (top) for RGR and LD heat map (bottom) of *q5.7*. The red dot indicates the lead SNP rs5_14379574. **(G)** The haplotypes in the 1 kb of the upstream promoter region and coding sequence region of *LOC_Os05g24770*. **(H)** The relative expression levels of *LOC_Os05g24770* under saline-alkali stress for 24 h and control conditions. Gene expression was normalized to that of the *UBQ* gene control. **(I)** The distribution of RGR for the three haplotypes of *LOC_Os05g24770*. **(J)** Frequency of three haplotypes of *LOC_Os05g24770* in subpopulations. Chi-square tests were used to determine significant differences in the haplotype proportions between different subpopulations in **(B, G)**, with ***P < 0.001. Different letters above the boxplot indicate significant differences among haplotypes according to Duncan’s multiple range *post-hoc* test (*P* < 0.05) in **(D, I)**. **P < 0.01 and ***P < 0.001 (two-tailed Student’s *t*-test) in **(C, H)**. n.s. indicates no significant difference (two-tailed Student’s t-test) in **(C, H)**. “*Geng*-S”, “*Geng*-T”, “*Xian*-S” and “*Xian*-T” in **(C, H)** represent saline-alkali-sensitive *Geng* accession (American Huangkedao), saline-alkali-tolerant *Geng* accession (SACHIKAZE), saline-alkali-sensitive *Xian* accession (ARC 6052) and saline-alkali-tolerant *Xian* accession (ARC 18597), respectively. The data of the relative expression levels in **(C, H)** are presented as mean ± SD (*n* = 3, biological replicates).

According to the criteria for candidate gene prediction (see Methods), we identified four and two candidate genes in the important main-effect QTNs, *q1.11* and *q5.7*, respectively (**Figure 2E**). *q1.11* was the most significant (*P* =5.10 × 10^-80^) QTN associated with RGR. The LD block region of *q1.11* was estimated from 25.820 to 25.866 Mb (46.3 kb interval) on chromosome 1 (**Figure 3A**). The lead SNP associated with RGR, rs1_25863302, is located in the promoter region of a putative auxin efflux carrier gene *LOC_Os01g45550* (*OsPIN3t*). Five major haplotypes (*n* ≥ 15 accessions) were detected using ten SNPs in the 1 kb upstream region of *LOC_Os01g45550* (**Figure 3B**). Among the five haplotypes, Hap4 with the largest mean RGR (0.85) was considered the favorable haplotype for saline-alkali tolerance (**Figure 3D**), which was present only in *Geng* accessions (**Figure 3E**). *OsPIN3t* was significantly up-regulated in saline-alkali-sensitive *Geng* accession (American Huangkedao) and saline-alkali-tolerant *Xian* accession (ARC 18597) under saline-alkali stress, whereas it was significantly down-regulated in saline-alkali-tolerant *Geng* accession (SACHIKAZE) under saline-alkali stress (**Figure 3C**). Interestingly, American Huangkedao carries the non-favorable haplotype Hap3 and SACHIKAZE carries the favorable haplotype Hap4. In contrast, *q5.7* was the most significant (*P* = 1.94 × 10^-30^) one associated with four of five saline-alkali tolerance traits (RGR, RGI, RVI, RSL and RRL) except RVI, while the *q3.6* (*P* = 4.71 × 10^-12^) and *q11.16* (*P* = 8.12 × 10^-13^) were the QTNs associated with four saline-alkali tolerance traits except RGI and RSL, respectively. The total phenotypic variance explained by this QTN was 2.69% for RGR, 4.68% for RGI, 3.43% for RSL, and 2.59% for RRL. The *q5.7* LD block region of lead SNP (rs5_14379574) associated with RGR was estimated from 14.206 to 14.406 Mb (200.0 kb interval) on chromosome 5 (**Figure 3F**). According to reported information of gene annotations (**Figure 2E**), we found that there was a potential salt-tolerant gene *LOC_Os05g24770* encoding a reticulon domain-containing protein in the LD block region. *LOC_Os05g24770* was significantly up-regulated in saline-alkali-sensitive *Geng* accession (American Huangkedao), while significantly down-regulated in saline-alkali-tolerant *Geng* accession (SACHIKAZE) under saline-alkali stress (**Figure 3H**). Three major haplotypes (*n* ≥ 15 accessions) of *LOC_Os05g24770* were detected based on 13 SNPs in its 1 kb upstream region and the coding sequence region (**Figure 3G**). Among the three haplotypes, Hap2 had the largest mean RGR of 0.82, while the mean RGR of Hap1 and Hap3 were 0.77 and 0.62 (**Figure 3I**). Thus, we defined Hap2 as the favorable haplotype for saline-alkali tolerance, which was enriched in *Xian* accessions (**Figure 3J**).

### 3.4 QQIs for rice saline-alkali tolerance at the germination stage

**Figure 4:**
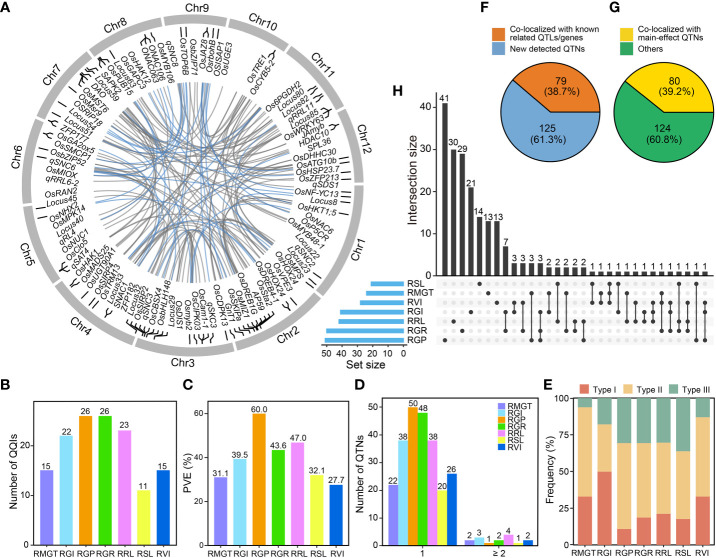
Characterization of QQIs’ contribution to the phenotypic variance of saline-alkali tolerance-related traits. **(A)** Circos plot shows the positions of the epistatic QTNs. The internal lines of the circos link the significant epistatic interactions between QTNs affecting the saline-alkali tolerance-related traits at the germination stage in rice. Blue line, PVE ≥ 3%; grey line, PVE < 3%. The known QTLs/genes related to saline-alkali tolerance that are co-localized with epistatic QTNs are marked. **(B)** The number of QQIs for different traits. **(C)** The total PVE for different traits. **(D)** Hotspots of epistatic QTNs. “1” on the X-axis indicates an epistatic QTN only involved in a pair of QQI. “≥ 2” on the X-axis indicates an epistatic QTN involved in at least two pairs of QQIs. **(E)** Frequency distribution of three types of QQI results for different traits. Type I, both two epistatic QTNs of a pair of QQI were identified with main-effect; Type II, one epistatic QTN of a pair of QQI was identified with main-effect; Type III, none of the two epistatic QTNs of a pair of QQI was identified with main-effect. **(F)** Different ratios for epistatic QTNs linked with known QTLs related to saline-alkali tolerance and genes related to saline-alkali tolerance in rice. Orange color, the number of known QTLs/genes in epistatic QTNs; blue color, the number of epistatic QTNs without any known QTLs/genes. **(G)** Different ratios for epistatic QTNs linked with main-effect QTNs. Yellow color, the number of main-effect QTNs in epistatic QTNs; green color, the number of epistatic QTNs without any main-effect QTNs. **(H)** Intersection size of epistatic QTNs between different traits.

Using the IIIVmrMLM software, we identified 134 significant QQI pairs (**Supplementary Table 4**; **Figure 4A**) for the saline-alkali tolerance-related traits between 204 epistatic QTNs (**Supplementary Table 5**), including 80 (39.2%) of the identified main-effect QTNs (**Figure 4G**). The identified QQI pairs (explained total trait phenotypic variance) were 26 (60.0%) for RGP, 15 (31.1%) for RMGT, 22 (39.5%) for RGI, 26 (43.6%) for RGR, 23 (47.0%) for RRL, 15 (27.7%) for RVI, and 11 (32.1%) for RSL, respectively (**Figures 4B, C** **)**. Most (90.5%) epistatic QTNs were involved in single pairwise epistasis, and were associated with only a single trait (**Figure 4D**). Interestingly, 33 and 10 epistatic QTNs associated with two and three traits, respectively (**Figure 4H**). This result indicated that most epistatic QTNs were highly specific regarding the QQIs and traits involved. Notably, 79 (38.7%) epistatic QTNs co-localized with the previously reported QTLs related to alkali tolerance and genes related to saline-alkali tolerance in rice (**Figure 4F**), while the QQI pair was detected between rs3_17097612 and rs7_8490713 with the largest PVE (11.3%) on RRL.

The QQIs detected in this study could be divided into three types: (I) QQIs occurred between two main-effect QTNs; (II) QQIs occurred between one main-effect QTN and one non-main-effect QTN; and (III) QQIs occurred between two non-main-effect QTNs. The proportion of different QQI types varied considerably across different traits (**Figure 4E**). Specifically, the proportion of type I QQIs was highest for RGI (50.0%), followed by RVI (33.3%), RMGT (33.3%), RRL (21.7%), RGR (19.2%), RSL (18.2%) and lowest (11.5%) in RGP. The frequency of type II QQIs was highest (60.0%) for RMGT, followed by RGP (57.7%), RVI (53.3%), RGR (50.0%), RRL (47.8%), RSL (45.5%) and lowest (31.8%) in RGI. In contrast, the proportion of type III QQIs was highest (36.4%) for RSL, followed by RGP (30.8%), RGR (30.8%), RRL (30.4%), RGI (18.2%), RVI (13.3%) and lowest (6.7%) in RMGT (**Figure 4E**).

### 3.5 Cases of important QQIs for the saline-alkali tolerance

**Figure 5:**
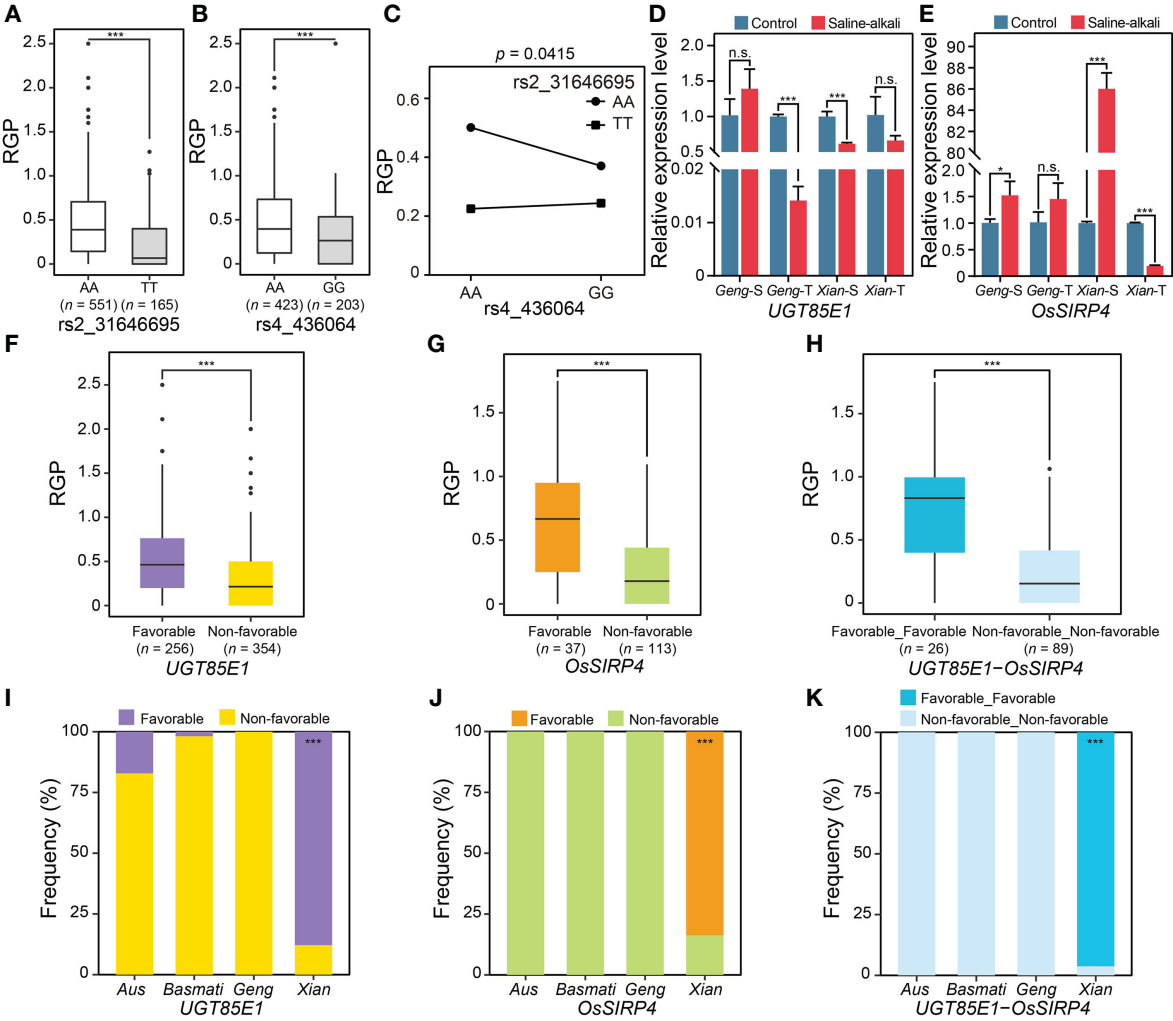
Characterization of an important QQI for RGP. Boxplots of RGP in different genotypes at two epistatic QTNs, rs2_31646695 **(A)** and rs4_436064 **(B)**. **(C)** Interaction plot for epistasis between two epistatic QTNs (rs2_31646695 × rs4_436064). **(D)** The expression levels of a candidate gene of epistatic QTN rs2_31646695, *UGT85E1*, under saline-alkali stress and control conditions. **(E)** The expression levels of a candidate gene of epistatic QTN rs4_436064, *OsSIRP4*, under saline-alkali stress and control conditions. Gene expression was normalized to that of the *UBQ* gene control. The phenotypic distribution of RGP with different major haplotypes for *UGT85E1* **(F)** and *OsSIRP4* **(G)**. **(H)** The RGP distribution for the major haplotype combinations of *UGT85E1* × *OsSIRP4*. “Favorable_Favorable” represents the haplotype combination of *UGT85E1* ^Favorable haplotype^ × *OsSIRP4* ^Favorable haplotype^. “Non-favorable_Non-favorable” represents the haplotype combination of *UGT85E1* ^Non-favorable haplotype^ × *OsSIRP4* ^Non-favorable haplotype^. Haplotype frequency distribution of *UGT85E1* **(I)** and *OsSIRP4* **(J)** in subpopulations. **(K)** Haplotype frequency of the major haplotype combinations of *UGT85E1* × *OsSIRP4* in subpopulations. *P < 0.05 and ***P < 0.001 (two-tailed Student’s *t*-test) in **(A, B, D–H)**. n.s. indicates no significant difference (two-tailed Student’s t-test) in **(D, E)**. Hypergeometric test was used to determine significant enrichment of a haplotype/haplotype combination in a specific subpopulation in **(I–K)**, with ***P < 0.001. Two-way ANOVA was used to determine the interaction between two QTNs in **(C)**. “*Geng*-S”, “*Geng*-T”, “*Xian*-S” and “*Xian*-T” in **(D, E)** represent saline-alkali sensitive *Geng* accession (American Huangkedao), saline-alkali tolerant *Geng* accession (SACHIKAZE), saline-alkali sensitive *Xian* accession (ARC 6052) and saline-alkali tolerant *Xian* accession (ARC 18597), respectively. The data of the relative expression levels in **(D, E)** are presented as mean ± SD (*n* ≥ 2, biological replicates).

Two important of QQIs with large PVE were noted. The first case was the QQI between rs2_31646695 and rs4_436064, which explained 4.43% of the phenotypic variation for RGP. The mean phenotypic values ​​of the two alleles (AA and TT) at rs2_31646695 were significantly different, with the mean RGP values ​​of AA and TT being 0.45 and 0.24, respectively (**Figure 5A**), while the phenotypic values of the two alleles (AA and GG) at rs4_436064 were also significantly different, with the mean RGP values ​​of AA and GG being 0.46 and 0.33, respectively (**Figure 5B**). The phenotype of the accessions with the non-favorable allele GG at rs4_436064 was further enhanced by the favorable allele AA at rs2_31646695 (i.e., the mean RGP increased from 0.24 in GGTT to 0.37 in GGAA) (**Figure 5C**). Based on the literature search and expression profiles under abiotic stress conditions, two promising candidate genes of the QQI between rs2_31646695 and rs4_436064 were identified, including an E3 ligase gene *LOC_Os04g01490* (*OsSIRP4*) near rs4_436064 and a glycosyltransferase gene *LOC_Os02g51900* (*UGT85E1*) near rs2_31646695. *UGT85E1* was significantly down-regulated in saline-alkali-tolerant *Geng* accession (SACHIKAZE) and saline-alkali-sensitive *Xian* accession (ARC 6052) under saline-alkali stress (**Figure 5D**). *OsSIRP4* was significantly up-regulated in saline-alkali-sensitive *Geng* accession (American Huangkedao) and saline-alkali-sensitive *Xian* accession (ARC 6052), while significantly down-regulated in the saline-alkali-tolerant *Xian* accession (ARC 18597) under saline-alkali stress (**Figure 5E**). Based on the gene-haplotype analysis using the SNPs in the 1 kb upstream region and the coding sequence region, we found significant differences in RGP between the favorable and non-favorable haplotypes of both *UGT85E1* and *OsSIRP4* (**Figures 5F, G** **)**. Moreover, two haplotype combinations of the two genes were obtained in the GWAS panel (**Figure 5H**). The favorable combination consisted of two favorable haplotypes of the two genes, with an average RGP value of 0.71 (**Figure 5H**). The favorable haplotype of *UGT85E1* was mainly enriched in population *Xian*, while the favorable haplotype of *OsSIRP4* and the favorable haplotype combination were carried only by *Xian* accessions (**Figures 5I–K**).

**Figure 6:**
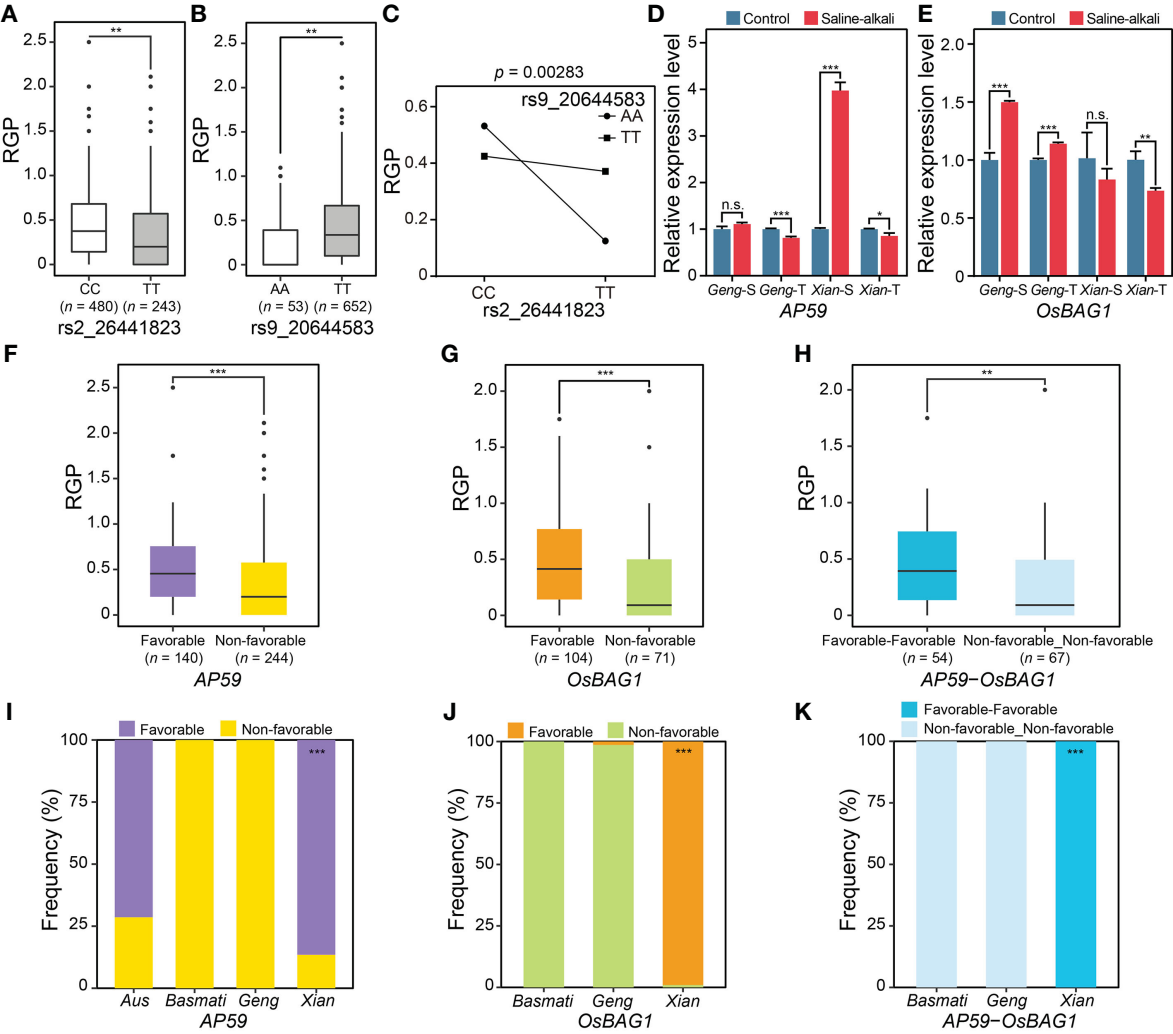
Characterization of an important QQI for RGP. **(A)** Boxplots of RGP in different genotypes at two epistatic QTNs, rs2_26441823 **(A)** and rs9_20644583 **(B)**. **(C)** Interaction plot for epistasis between two QTNs (rs2_26441823 × rs9_20644583). **(D)** The expression levels of a candidate gene of epistatic QTN rs2_26441823, *AP59*, under saline-alkali stress and control conditions. **(E)** The expression levels of a candidate gene of epistatic QTN rs9_20644583, *OsBAG1*, under saline-alkali stress and control conditions. Gene expression was normalized to that of the *UBQ* gene control. The distribution of RGP for the different major haplotypes for *AP59* **(F)** and *OsBAG1* **(G)**. **(H)** The distribution of RGP for the two major haplotype combinations of *AP59* × *OsBAG1*. “Favorable_Favorable” represents the haplotype combination of *AP59* ^Favorable haplotype^ × *OsBAG1* ^Favorable haplotype^. “Non-favorable_Non-favorable” represents the haplotype combination of *AP59* ^Non-favorable haplotype^ × *OsBAG1* ^Non-favorable haplotype^. Haplotype frequency distribution of *AP59* **(I)** and *OsBAG1* **(J)** in subpopulations. **(K)** Frequency of the major haplotype combinations of *AP59* × *OsBAG1* in subpopulations. *P < 0.05, **P < 0.01 and ***P < 0.001 (two-tailed Student’s *t*-test) in **(A, B, D–H)**. n.s. indicates no significant difference (two-tailed Student’s t-test) in **(D, E)**. Hypergeometric test was used to determine significant enrichment of a haplotype/haplotype combination in a specific subpopulation in **(I–K)**, with ***P < 0.001. Two-way ANOVA was used to determine the interaction between two QTNs in **(C)**. “*Geng*-S”, “*Geng*-T”, “*Xian*-S” and “*Xian*-T” in **(D)** and **(E)** represent saline-alkali-sensitive *Geng* accession (American Huangkedao), saline-alkali-tolerant *Geng* accession (SACHIKAZE), saline-alkali-sensitive *Xian* accession (ARC 6052) and saline-alkali-tolerant *Xian* accession (ARC 18597), respectively. The data of the relative expression levels in **(D, E)** are presented as mean ± SD (*n* = 3, biological replicates).

The second case was the QQI between rs2_26441823 and rs9_20644583, explaining 4.52% of the phenotypic variation of RGP. The phenotypic values ​​of the two alleles (CC and TT) of rs2_26441823 were significantly different, with the mean RGP values ​​of CC and TT being 0.43 and 0.34, respectively (**Figure 6A**), while the phenotypic values of the two alleles (AA and TT) at rs9_20644583 were also significantly different, with the mean RGP values ​​of AA and TT being 0.24 and 0.41, respectively (**Figure 6B**). The phenotype of the accessions with the non-favorable allele TT at rs2_26441823 was further enhanced by the favorable allele TT at rs9_20644583 (i.e., the mean RGP increased from 0.12 in TTAA to 0.37 in TTTT) (**Figure 6C**). Based on the literature search and expression profiles under abiotic stress conditions, two promising candidate genes of the QQI between rs2_26441823 and rs9_20644583 were identified, including an ethylene-responsive transcriptional factor gene *LOC_Os02g43790* (*AP59*/*OsBIERF3*) near rs2_26441823 and a Bcl-2-associated athanogene protein gene *LOC_Os09g35630* (*OsBAG1*) near rs9_20644583. *AP59* was significantly down-regulated in saline-alkali-tolerant *Geng* accession (SACHIKAZE) and saline-alkali-tolerant *Xian* accession (ARC 18597) under saline-alkali stress, while significantly up-regulated in saline-alkali-sensitive *Xian* accession (ARC 6052) under saline-alkali stress (**Figure 6D**). *OsBAG1* was significantly up-regulated in saline-alkali-sensitive *Geng* accession (American Huangkedao) and saline-alkali-tolerant *Geng* accession (SACHIKAZE) under saline-alkali stress, while significantly down-regulated in the saline-alkali-tolerant *Xian* accession (ARC 18597) under saline-alkali stress (**Figure 6E**). Significant differences in RGP were both observed between the favorable and non-favorable haplotypes of *AP59* and *OsBAG1* (**Figures 6F, G** **)**. Among the two haplotype combinations of the two genes in the GWAS panel (**Figure 6H**), the favorable saline-alkali tolerant combination was made up of the two favorable haplotypes from the two genes, with an average RGP value of 0.46 (**Figure 6H**). The favorable haplotypes of *AP59* and *OsBAG1* were mainly carried by *Xian* accessions (**Figures 6I, J** **)**, while the favorable haplotype combination was only enriched in population *Xian* (**Figure 6K**).

### 3.6 Application of epistatic QTNs for genomic prediction

**Figure 7:**
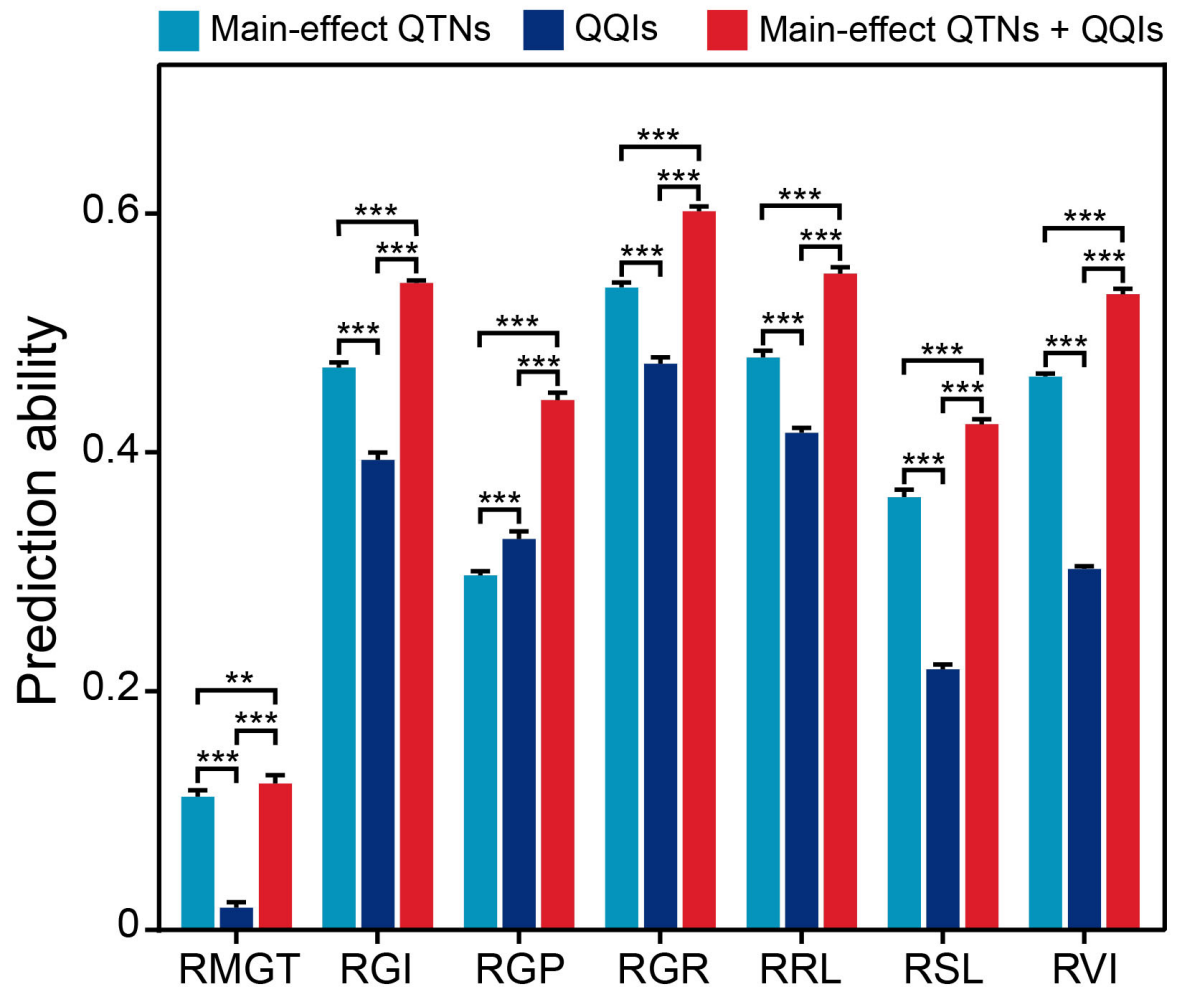
Comparison of prediction abilities (*r* ^2^) among three SNP datasets for different traits based on the 10-fold cross-validation method. ** and *** indicate significant differences at *P* < 0.01 and *P* < 0.001 (two-sided Wilcoxon rank-sum test), respectively.

To determine whether the identified epistatic QTNs have advantages for genomic prediction of saline-alkali tolerance, we compared the performances of the main-effect QTNs, the epistatic QTNs and their combined datasets in genomic prediction for the seven saline-alkali tolerance-related traits using ridge regression best linear unbiased prediction (rrBLUP) approaches. Based on 10 times repeated 10-fold cross-validations, the main-effect QTN dataset showed significantly higher prediction abilities than the epistatic QTN dataset for all traits except for RGP (**Figure 7**). Notably, the combined dataset of main-effect and epistatic QTNs showed significantly higher prediction abilities than either main-effect or epistatic QTNs alone for all traits. In other words, the prediction abilities of the seven traits based on the main-effect QTNs were significantly improved by inclusion of the epistatic QTNs, with an average increased power of 7.0%, when the epistatic QTNs were included in the prediction model. Among the seven traits, the prediction ability of RGP was improved most (with an increase of 14.7%) by the epistatic QTNs compared to the prediction ability by the main-effect QTNs, while the prediction ability of RMGT was improved least (with an increase of 1.1%) (**Figure 7**).

## 4 Discussion

### 4.1 Epistasis is an important component in the genetic architecture of saline-alkali tolerance in rice

As the global climate changes, soil saline-alkalization has become an increasingly serious threat to world crop production and food security. To facilitate the development of rice cultivars with improved saline-alkali tolerance, we aimed to dissect the genetic architecture of rice saline-alkali tolerance using a modified strategy of GWAS. The identification of 165 main-effect QTNs and 134 QQIs that explained an average of 52.6% and 40.1% total PVE for each of the rice saline-alkali tolerance traits during germination was consistent with the current knowledge that rice saline-alkali tolerance is a complex trait involving large numbers of loci and multiple genetic mechanisms (Zhang et al., 2013). Of these main-effect QTNs, 70 (42.4%) overlapped with previously reported QTLs/genes related to saline-alkali tolerance in rice, indicating a very low false positive rate in QTN detection of this study. Moreover, we identified two promising candidate genes, *LOC_Os01g45550* (*OsPIN3t*) and *LOC_Os05g24770*, associated with saline-alkali tolerance at the germination stage in rice. *LOC_Os01g45550* (*OsPIN3t*) is involved in drought stress response in rice and acts in auxin polar transport (Zhang et al., 2012). Both drought and saline-alkali stresses are known to cause osmotic stress in plant root cells. Also, salt stress is known to significantly impact auxin transport in plants, which is expected to influence plant root development through its effect on auxin distribution and epidermal biosynthesis (Korver et al., 2018). *LOC_Os05g24770* encodes a reticulon domain-containing protein in rice. The reticulon domain-containing protein gene, *F2DHE3* in Tibetan wild barley was reportedly involved in salt adaptation of the wild barley as it was up-regulated in the variety under high Na^+^ accumulation (Shen et al., 2016). Furthermore, another reticulon family protein gene, *RTNLB2*, in Arabidopsis was also up-regulated under salt stress (Ma and Bohnert, 2007). In both cases, *OsPIN3t* and *LOC_Os05g24770* are suggested to be involved in saline-alkali tolerance, but how they are contributing to saline-alkali tolerance remains to be experimentally validated.

One major discovery of this study was the importance of epistasis in determining rice saline-alkali tolerance. Although epistasis is known to be an important genetic basis of complex traits (Yu et al., 1997; Li et al., 2001; Marchini et al., 2005; Evans et al., 2006; Wei et al., 2014), it remains a huge challenge to detect epistasis in natural populations by GWAS (Manicacci et al., 2009; Wurschum et al., 2011; Zhang et al., 2015; Wen et al., 2016; Luo et al., 2017). This is because the power in detecting QTNs, particularly epistatic QTNs, is influenced much more by the population structure of the sampled accessions in a GWAS study, which results from the presence of multiple alleles at virtually all loci in a random germplasm population, and varied frequencies of different alleles and multi-locus genotypes at specific loci across different populations of the sample accessions. Fortunately, the newly developed method by Li et al., 2022a; Li et al., 2022b made it possible to detect epistasis by GWAS. For the two cases of important QQIs with large PVE (**Figures 5**, **6**), we identified four promising candidate genes. For the first case of important QQI between rs2_31646695 and rs4_436064, *LOC_Os02g51900* (*UGT85E1*) and *LOC_Os04g01490* (*OsSIRP4*) were identified as the promising candidate genes. *OsSIRP4*, highly induced under salt stress in rice, acts as a negative regulator in the plant response to salt stress via the 26S proteasomal system regulation of substrate proteins, OsPEX11-1, which provides important information for the adaptation and regulation in rice (Kim and Jang, 2021). The expression of *UGT85E1* was significantly up-regulated by drought stress and ABA treatments, while its overexpression led to the enhanced tolerance in transgenic rice plants to drought stress (Liu et al., 2021a). Salt or drought stress generates osmotic stress in plant cells and dramatically increases cellular ABA levels (Fernando and Schroeder, 2016). For the second case of important QQI between rs2_26441823 and rs9_20644583, *LOC_Os02g43790* (*AP59*/*OsBIERF3*) and *LOC_Os09g35630* (*OsBAG1*) were identified as the promising candidate genes. *AP59*/*OsBIERF3* was up-regulated by salt, cold, drought and wounding in rice (Cao et al., 2006). The overexpression of *AP59* in rice under the control of the constitutive promoter *OsCc1* increased the tolerance to drought and high salinity at the vegetative stage (Oh et al., 2009). *OsBAG1* was up-regulated (at least four-fold) after continuous high-temperature treatment and was down-regulated in two varieties (Dagad deshi and IR20) under drought stress (Zhou et al., 2021). As heat, drought and salt stress are the main abiotic stresses, we speculate that *OsBAG1* may also respond to salt stress. The function of these promising candidate genes of important QQIs should be experimentally validated in future.

In fact, this study, to our knowledge, was the first effort to detect genome-wide epistasis for saline-alkali tolerance by GWAS using natural rice germplasm populations. Nevertheless, two interesting observations were noted regarding how to detect epistasis using GWAS of natural populations and the interpretation of the detected epistatic QTNs. Firstly, we detected more epistatic QTNs than main-effect QTNs, and 79 (38.7%) of the identified epistatic QTNs overlapped with previously reported QTLs/genes related to saline-alkali tolerance in rice, indicating a very low false positive rate in the epistatic QTNs and the reliability of the method used in this study, even though most of the identified QQIs appeared to have smaller effects than the main-effect QTNs. Secondly, significantly more (39.2%) main-effect QTNs were involved in QQIs in this study than in previous reports from segregating populations of bi-parental crosses in which most QQIs occur between QTNs that do not have detectable main-effects (Li et al., 1997; Li et al., 2001). Theoretically, this was expected because most QQIs were undetectable in natural germplasm populations due to the fact that the haplotype combination frequencies of any specific pair of QQI in the 3KRG were much lower than their Mendelian segregation frequencies in bi-parental segregating populations (**Figures 5K**, **6K**). The impact of the population structure on the power in detecting epistasis is particularly more pronounced in inbreeding species such as rice than in outcrossing ones (Doust et al., 2014). Thus, the epistatic QTNs affecting saline-alkali resistance detected in this study were important ones, which can be verified genetically using bi-parental or multiple-parent mapping populations with balanced frequencies of bi-allelic or multi-locus genotypes in which the epistatic variance is maximized (Mackay, 2014; Sakai et al., 2021; Wang et al., 2022). More importantly, molecular validation of epistatic QTNs identified by GWAS will be facilitated by the higher resolution of their candidate genes, and by their functional relationships of the candidate genes, i.e., to experimentally validate if there is one-way (regulating or regulated) or mutual functional dependency between the interacting QTN candidates (Zhang et al., 2011). Taking together, our results supported that epistasis is an important genetic basis of saline-alkali tolerance in rice.

### 4.2 Breeding application of the epistatic QTNs

The ultimate goal of past theoretical work of rice, including both genetic dissection of complex traits and functional genomic research aiming at cloning and functional characterization of genes controlling complex traits, is to apply these results to accelerate genetic gains in improving complex traits in future breeding efforts. One common strategy is to select and pyramid favorable alleles at many target loci to improve traits such as yield potential and abiotic stress tolerances in breeding. It is also clear that virtually all complex traits are controlled by complex signaling pathways involving large numbers of loci. However, the presence of epistasis would suggest that it is unreliable to predict the phenotypic effects of breeding progeny based on their genotypes at single loci. Thus, it is crucial to discover the epistatic interactions between genes to optimize gene pyramiding experiments (Yan et al., 2011). In these cases, taking both the additive and epistatic effects of alleles among multiple genes into consideration would allow identification of the most favorable allele combination(s) at a specific set of epistatic loci with maximum effects on target traits, which otherwise can’t be achieved through simply pyramiding multiple alleles at single loci (Benfey and Mitchell-Olds, 2008). This was well demonstrated in our genome-wide selection experiments in which inclusion of both main-effect and epistatic QTNs had consistently higher predictability for all saline-alkali resistance traits. Similar results were achieved in an effort to improve rice saline-alkali tolerance at the germination stage by pyramiding the favorable haplotypes of multiple saline-alkali-tolerant main-effect QTNs and creating the optimal haplotype combinations (Mei et al., 2022). Obviously, our results suggest that the identified epistatic QQIs and their frequency distribution in different subpopulations can provide valuable information for molecular breeding for improving complex traits including rice saline-alkali tolerance. Thus, it is now possible to enhance saline-alkali tolerance of saline-alkali sensitive rice varieties by introgressing the favorable haplotype combinations via marker-assisted selection and chip-based genotyping. For example, the favorable haplotype combinations, *UGT85E1* ^Hap1^ × *OsSIRP4* ^Hap2^ and *AP59* ^Hap1^ × *OsBAG1* ^Hap1^ of the epistatic QQIs can be used to improve RGP by selective introgression (**Supplementary Table 6**). Clearly, the genetic information of the germplasm resources carrying favorable haplotype combinations obtained in this study would provide useful information for selecting suitable genetic materials for further functional genomic research, though their values for improving rice saline-alkali tolerance should be actively tested in real breeding efforts in future.

## 5 Conclusion

Through high-density SNPs, extensive phenotyping of the large-scale rice natural population under the saline-alkali stress and control conditions, and recent advancement in GWAS methodologies, we were able to conduct more robust and efficient genetic analysis of rice saline-alkali tolerance to better address QQI effects. Our results suggest that rice saline-alkali tolerance is a very complex trait with epistasis as an important genetic basis. The epistatic QQIs and their frequency distribution in germplasm resources can provide valuable information for future molecular breeding for improving rice saline-alkali tolerance at the germination stage.

## Data availability statement

The original contributions presented in the study are included in the article/**Supplementary Material**. Further inquiries can be directed to the corresponding authors.

## Author contributions

FZ and YS conceived, designed the experiments; GZ, JJ, JL, KL, DB, XW and XYZ performed the experiments; GZ, ZB, ML, XQZ, WW and JX performed analysis and interpretation of the data; GZ, FZ and ZL wrote the manuscript; FZ revised the manuscript. All authors contributed to the article and approved the submitted version.

## References

[B1] Alexandrov N. Tai S. Wang W. Mansueto L. Palis K. Fuentes R. R.. (2015). SNP-seek database of SNPs derived from 3000 rice genomes. Nucleic Acids Res. 43 (Database issue), D1023–D1027. doi: 10.1093/nar/gku1039 25429973 PMC4383887

[B2] Alvarado A. D. Bradford K. J. Hewitt J. D. (1987). Osmotic priming of tomato seeds: effects on germination, field emergence, seedling growth, and fruit yield. J. Am. Soc. Hortic. Sci. 112 (3), 427–432. doi: 10.1126/science.1153716

[B3] Benfey P.N. Mitchell-Olds T. (2008). From genotype to phenotype: systems biology meets natural variation. Science 320 (5875), 495–497. doi: 10.1126/science.1153716 PMC2727942 18436781

[B4] Brachi B. Morris G. P. Borevitz J. O. (2011). Genome-wide association studies in plants: the missing heritability is in the field. Genome Biol. 12 (10), 232. doi: 10.1186/gb-2011-12-10-232 22035733 PMC3333769

[B5] Campbell S. A. Nishio J. N. (2000). Iron deficiency studies of sugar beet using an improved sodium bicarbonate-buffered hydroponic growth system. J. Plant Nutr. 23 (6), 741–757. doi: 10.1080/01904160009382056

[B6] Carré C. Carluer J.B. Chaux C. Roche N. Mas A. Krouk G. (2022). Full epistatic interaction maps retrieve part of missing heritability and improve phenotypic prediction. bioRxiv 2022-07. doi: 10.1101/2022.07.20.500572 PMC10962106 38523316

[B7] Cao Y. Song F. Goodman R. M. Zheng Z. (2006). Molecular characterization of four rice genes encoding ethylene-responsive transcriptional factors and their expressions in response to biotic and abiotic stress. J. Plant Physiol. 163 (11), 1167–1178. doi: 10.1101/2022.07.20.500572 16436304

[B8] Dai L. Li P. Li Q. Leng Y. Zeng D. Qian Q. (2022). Integrated multi-omics perspective to strengthen the understanding of salt tolerance in rice. Int. J. Mol. Sci. 23 (9), 5236. doi: 10.3390/ijms23095236 35563627 PMC9105537

[B9] Dong S. S. He W. M. Ji J. J. Zhang C. Guo Y. Yang T. L. (2021). LDBlockShow: a fast and convenient tool for visualizing linkage disequilibrium and haplotype blocks based on variant call format files. Brief Bioinform. 22 (4), bbaa227. doi: 10.1093/bib/bbaa227 33126247

[B10] Doust A. N. Lukens L. Olsen K. M. Mauro-Herrera M. Meyer A. Rogers K. (2014). Beyond the single gene: how epistasis and gene-by-environment effects influence crop domestication. Proc. Natl. Acad. Sci. 111 (17), 6178–6183. doi: 10.1073/pnas.1308940110 24753598 PMC4035984

[B11] Endelman J. B. (2011). Ridge regression and other kernels for genomic selection with r package rrBLUP. Plant Genome 4 (3), 250–255. doi: 10.3835/plantgenome2011.08.0024

[B12] Evans D. M. Marchini J. Morris A. P. Cardon L. R. (2006). Two-stage two-locus models in genome-wide association. PloS Genet. 2 (9), e157. doi: 10.1371/journal.pgen.0020157 17002500 PMC1570380

[B13] Fang S. Hou X. Liang X. (2021). Response mechanisms of plants under saline-alkali stress. Front. Plant Sci. 12. doi: 10.3389/fpls.2021.667458 PMC8213028 34149764

[B14] Fernando V. C. D. Schroeder D. F. (2016). “Role of ABA in arabidopsis salt, drought, and desiccation tolerance,” in Abiotic and biotic stress in plants - recent advances and future perspectives. (Rijeka, Croatia: IntechOpen). doi: 10.5772/61957

[B15] Ganapati R. K. Naveed S. A. Zafar S. Wang W. Xu J. (2022). Saline-alkali tolerance in rice: physiological response, molecular mechanism, and QTL identification and application to breeding. Rice Sci. 29 (5), 412–434. doi: 10.1016/j.rsci.2022.05.002

[B16] Gao C. Wang Y. Liu G. Yang C. Jiang J. Li H. (2008). Expression profiling of salinity-alkali stress responses by large-scale expressed sequence tag analysis in tamarix hispid. Plant Mol. Biol. 66 (3), 245–258. doi: 10.1007/s11103-007-9266-4 18058243

[B17] Gu Z. Gu L. Eils R. Schlesner M. Brors B. (2014). Circlize implements and enhances circular visualization in r. Bioinformatics 30 (19), 2811–2812. doi: 10.1093/bioinformatics/btu393 24930139

[B18] Guo M. Wang R. Wang J. Hua K. Wang Y. Liu X.. (2014). ALT1, a Snf2 family chromatin remodeling ATPase, negatively regulates alkaline tolerance through enhanced defense against oxidative stress in rice. PloS One 9 (12), e112515. doi: 10.1371/journal.pone.0112515 25473841 PMC4256374

[B19] He Y. Yang B. He Y. Zhan C. Cheng Y. Zhang J.. (2019). A quantitative trait locus, qSE3, promotes seed germination and seedling establishment under salinity stress in rice. Plant J. 97 (6), 1089–1104. doi: 10.1111/tpj.14181 30537381 PMC6850641

[B20] Huang X. Y. Chao D. Y. Gao J. P. Zhu M. Z. Shi M. Lin H. X. (2009). A previously unknown zinc finger protein, DST, regulates drought and salt tolerance in rice via stomatal aperture control. Genes Dev. 23 (15), 1805–1817. doi: 10.1101/gad.1812409 19651988 PMC2720257

[B21] Huang F. Jiang Y. Chen T. Li H. Fu M. Wang Y.. (2022). New data and new features of the FunRiceGenes (Functionally characterized rice genes) database: 2021 update. Rice (N Y) 15 (1), 23. doi: 10.1186/s12284-022-00569-1 35438356 PMC9018921

[B22] Huang R. Jiang L. Zheng J. Wang T. Wang H. Huang Y.. (2013). Genetic bases of rice grain shape: so many genes, so little known. Trends Plant Sci. 18 (4), 218–226. doi: 10.1016/j.tplants.2012.11.001 23218902

[B23] Huang X. Wei X. Sang T. Zhao Q. Feng Q. Zhao Y.. (2010). Genome-wide association studies of 14 agronomic traits in rice landraces. Nat. Genet. 42 (11), 961–967. doi: 10.1038/ng.695 20972439

[B24] Kaiwen G. Zisong X. Yuze H. Qi S. Yue W. Yanhui C.. (2020). Effects of salt concentration, pH, and their interaction on plant growth, nutrient uptake, and photochemistry of alfalfa (Medicago sativa) leaves. Plant Signal Behav. 15 (12), 1832373. doi: 10.1080/15592324.2020.1832373 33073686 PMC7671061

[B25] Kawahara Y. de la Bastide M. Hamilton J. P. Kanamori H. McCombie W. R. Ouyang S.. (2013). Improvement of the oryza sativa nipponbare reference genome using next generation sequence and optical map data. Rice 6 (1), 4. doi: 10.1186/1939-8433-6-4 24280374 PMC5395016

[B26] Kim J. H. Jang C. S. (2021). E3 ligase, the oryza sativa salt-induced RING finger protein 4 (OsSIRP4), negatively regulates salt stress responses via degradation of the OsPEX11-1 protein. Plant Mol. Biol. 105 (3), 231–245. doi: 10.1007/s11103-020-01084-x 33079323

[B27] Korte A. Farlow A. (2013). The advantages and limitations of trait analysis with GWAS: a review. Plant Methods 9 (1), 29. doi: 10.1186/1746-4811-9-29 23876160 PMC3750305

[B28] Korver R. A. Koevoets I. T. Testerink C. (2018). Out of shape during stress: a key role for auxin. Trends Plant Sci. 23 (9), 783–793. doi: 10.1016/j.tplants.2018.05.011 29914722 PMC6121082

[B29] Li Z. K. Luo L. J. Mei H. W. Wang D. L. Shu Q. Y. Tabien R.. (2001). Overdominant epistatic loci are the primary genetic basis of inbreeding depression and heterosis in rice. i. biomass and grain yield. Genetics 158 (4), 1755–1771. doi: 10.1093/genetics/158.4.1755 11514460 PMC1461757

[B30] Li Z. K. Pinson S. R. Park W. D. Paterson A. H. Stansel J. W. (1997). Epistasis for three grain yield components in rice (Oryza sativa l.). Genetics 145 (2), 453–465. doi: 10.1093/genetics/145.2.453 9071598 PMC1207809

[B31] Li J. Pu L. Han M. Zhu M. Zhang R. Xiang Y. (2014). Soil salinization research in China: advances and prospects. J. Geograph. Sci. 24 (5), 943–960. doi: 10.1007/s11442-014-1130-2

[B32] Li M. Zhang Y. W. Xiang Y. Liu M. H. Zhang Y. M. (2022a). IIIVmrMLM: the r and c++ tools associated with 3VmrMLM, a comprehensive GWAS method for dissecting quantitative traits. Mol. Plant 15 (8), 1251–1253. doi: 10.1016/j.molp.2022.06.002 35684963

[B33] Li M. Zhang Y. W. Zhang Z. C. Xiang Y. Liu M. H. Zhou Y. H.. (2022b). A compressed variance component mixed model for detecting QTNs and QTN-by-environment and QTN-by-QTN interactions in genome-wide association studies. Mol. Plant 15 (4), 630–650. doi: 10.1016/j.molp.2022.02.012 35202864

[B34] Li N. Zheng H. Cui J. Wang J. Liu H. Sun J.. (2019). Genome-wide association study and candidate gene analysis of alkalinity tolerance in japonica rice germplasm at the seedling stage. Rice (N Y) 12 (1), 24. doi: 10.1186/s12284-019-0285-y 30976929 PMC6459459

[B35] Li X. Zheng H. Wu W. Liu H. Wang J. Jia Y.. (2020). QTL mapping and candidate gene analysis for alkali tolerance in japonica rice at the bud stage based on linkage mapping and genome-wide association study. Rice (N Y) 13 (1), 48. doi: 10.1186/s12284-020-00412-5 32676742 PMC7364718

[B36] Liu Q. Dong G. R. Ma Y. Q. Zhao S. M. Liu X. Li X. K.. (2021a). Rice glycosyltransferase gene UGT85E1 is involved in drought stress tolerance through enhancing abscisic acid response. Front. Plant Sci. 12. doi: 10.3389/fpls.2021.790195 PMC8733621 35003178

[B37] Liu Q. Lan G. Zhu Y. Chen K. Shen C. Zhao X.. (2021b). Genome-wide association study on resistance to rice black-streaked dwarf disease caused by rice black-streaked dwarf virus. Plant Dis. 105 (3), 607–615. doi: 10.1094/PDIS-10-19-2263-RE 32830595

[B38] Liu H. J. Yan J. (2019). Crop genome-wide association study: a harvest of biological relevance. Plant J. 97 (1), 8–18. doi: 10.1111/tpj.14139 30368955

[B39] Livak K. J. Schmittgen T. D. (2001). Analysis of relative gene expression data using real-time quantitative PCR and the 2–ΔΔCT method. Methods 25 (4), 402–408. doi: 10.1006/meth.2001.1262 11846609

[B40] Luo X. Ding Y. Zhang L. Yue Y. Snyder J. H. Ma C.. (2017). Genomic prediction of genotypic effects with epistasis and environment interactions for yield-related traits of rapeseed (Brassica napus l.). Front. Genet. 8. doi: 10.3389/fgene.2017.00015 PMC5318398 28270831

[B41] Ma S. Bohnert H. J. (2007). Integration of arabidopsis thaliana stress-related transcript profiles, promoter structures, and cell-specific expression. Genome Biol. 8 (4), R49. doi: 10.1186/gb-2007-8-4-r49 17408486 PMC1896000

[B42] Mackay T. F. C. (2014). Epistasis and quantitative traits: using model organisms to study gene–gene interactions. Nat. Rev. Genet. 15 (1), 22–33. doi: 10.1038/nrg3627 24296533 PMC3918431

[B43] Manicacci D. Camus-Kulandaivelu L. Fourmann M. Arar C. Barrault S. Rousselet A.. (2009). Epistatic interactions between Opaque2 transcriptional activator and its target gene CyPPDK1 control kernel trait variation in maize. Plant Physiol. 150 (1), 506–520. doi: 10.1104/pp.108.131888 19329568 PMC2675748

[B44] Marchini J. Donnelly P. Cardon L. R. (2005). Genome-wide strategies for detecting multiple loci that influence complex diseases. Nat. Genet. 37 (4), 413–417. doi: 10.1038/ng1537 15793588

[B45] Maurer A. Draba V. Jiang Y. Schnaithmann F. Sharma R. Schumann E.. (2015). Modelling the genetic architecture of flowering time control in barley through nested association mapping. BMC Genomics 16, 290. doi: 10.1186/s12864-015-1459-7 25887319 PMC4426605

[B46] Mei S. Zhang G. Jiang J. Lu J. Zhang F. (2022). Combining genome-wide association study and gene-based haplotype analysis to identify candidate genes for alkali tolerance at the germination stage in rice. Front. Plant Sci. 13. doi: 10.3389/fpls.2022.887239 PMC9033254 35463411

[B47] Munns R. Tester M. (2008). Mechanisms of salinity tolerance. Annu. Rev. Plant Biol. 59, 651–681. doi: 10.1146/annurev.arplant.59.032607.092911 18444910

[B48] Oh S.-J. Kim Y. S. Kwon C.-W. Park H. K. Jeong J. S. Kim J.-K. (2009). Overexpression of the transcription factor AP37 in rice improves grain yield under drought conditions. Plant Physiol. 150 (3), 1368–1379. doi: 10.1104/pp.109.137554 19429605 PMC2705040

[B49] Purcell S. Neale B. Todd-Brown K. Thomas L. Ferreira M. A. Bender D.. (2007). PLINK: a tool set for whole-genome association and population-based linkage analyses. Am. J. Hum. Genet. 81 (3), 559–575. doi: 10.1086/519795 17701901 PMC1950838

[B50] Quan R. Wang J. Hui J. Bai H. Lyu X. Zhu Y.. (2017). Improvement of salt tolerance using wild rice genes. Front. Plant Sci. 8. doi: 10.3389/fpls.2017.02269 PMC5776132 29387076

[B51] Rao P. S. Mishra B. Gupta S. R. (2013). Effects of soil salinity and alkalinity on grain quality of tolerant, semi-tolerant and sensitive rice genotypes. Rice Sci. 20 (4), 284–291. doi: 10.1016/s1672-6308(13)60136-5

[B52] Ren Z. H. Gao J. P. Li L. G. Cai X. L. Huang W. Chao D. Y.. (2005). A rice quantitative trait locus for salt tolerance encodes a sodium transporter. Nat. Genet. 37 (10), 1141–1146. doi: 10.1038/ng1643 16155566

[B53] Sabouri H. Sabouri A. (2008). New evidence of QTLs attributed to salinity tolerance in rice. Afr J. Biotechnol. 7 (24), 4376–4383. doi: 10.5897/AJB08.667

[B54] Sabouri H. Rezai A. M. Moumeni A. Kavousi A. Katouzi M. Sabouri A. (2009). QTLs mapping of physiological traits related to salt tolerance in young rice seedlings. Biol. plantarum 53 (4), 657–662. doi: 10.1007/s10535-009-0119-7

[B55] Sakai T. Abe A. Shimizu M. Terauchi R. (2021). RIL-StEp: epistasis analysis of rice recombinant inbred lines reveals candidate interacting genes that control seed hull color and leaf chlorophyll content. G3 (Bethesda) 11 (7), jkab130. doi: 10.1093/g3journal/jkab130 33871605 PMC8496299

[B56] Shabala S. (2013). Learning from halophytes: physiological basis and strategies to improve abiotic stress tolerance in crops. Ann. Bot. 112 (7), 1209–1221. doi: 10.1093/aob/mct205 24085482 PMC3806534

[B57] Shen Q. Fu L. Dai F. Jiang L. Zhang G. Wu D. (2016). Multi-omics analysis reveals molecular mechanisms of shoot adaption to salt stress in Tibetan wild barley. BMC Genomics 17 (1), 889. doi: 10.1186/s12864-016-3242-9 27821058 PMC5100661

[B58] Shi Y. Gao L. Wu Z. Zhang X. Wang M. Zhang C.. (2017). Genome-wide association study of salt tolerance at the seed germination stage in rice. BMC Plant Biol. 17 (1), 92. doi: 10.1186/s12870-017-1044-0 28558653 PMC5450148

[B59] Shi D. Wang D. (2005). Effects of various salt-alkaline mixed stresses on aneurolepidium chinense (Trin.) kitag. Plant Soil 271 (1-2), 15–26. doi: 10.1007/s11104-004-1307-z

[B60] Takagi H. Tamiru M. Abe A. Yoshida K. Uemura A. Yaegashi H.. (2015). MutMap accelerates breeding of a salt-tolerant rice cultivar. Nat. Biotechnol. 33 (5), 445–449. doi: 10.1038/nbt.3188 25798936

[B61] Thomson M. J. de Ocampo M. Egdane J. Rahman M. A. Sajise A. G. Adorada D. L.. (2010). Characterizing the saltol quantitative trait locus for salinity tolerance in rice. Rice 3 (2-3), 148–160. doi: 10.1007/s12284-010-9053-8

[B62] Wang Z. Cheng J. Chen Z. Huang J. Bao Y. Wang J.. (2012). Identification of QTLs with main, epistatic and QTL x environment interaction effects for salt tolerance in rice seedlings under different salinity conditions. Theor. Appl. Genet. 125 (4), 807–815. doi: 10.1007/s00122-012-1873-z 22678666

[B63] Wang W. Mauleon R. Hu Z. Chebotarov D. Tai S. Wu Z.. (2018b). Genomic variation in 3,010 diverse accessions of Asian cultivated rice. Nature 557 (7703), 43–49. doi: 10.1038/s41586-018-0063-9 29695866 PMC6784863

[B64] Wang M. Qi Z. Thyssen G. N. Naoumkina M. Jenkins J. N. McCarty J. C.. (2022). Genomic interrogation of a MAGIC population highlights genetic factors controlling fiber quality traits in cotton. Commun. Biol. 5 (1), 60. doi: 10.1038/s42003-022-03022-7 35039628 PMC8764025

[B65] Wang H. Takano T. Liu S. (2018a). Screening and evaluation of saline–alkaline tolerant germplasm of rice (Oryza sativa l.) in soda saline–alkali soil. Agronomy 8 (10), 205. doi: 10.3390/agronomy8100205

[B66] Wang Z.-f. Wang J.-f. Bao Y.-m. Wu Y.-y. Su X. Zhang H.-s. (2010). Inheritance of rice seed germination ability under salt stress. Rice Sci. 17 (2), 105–110. doi: 10.1016/s1672-6308(08)60112-2

[B67] Wang B. Xie G. Liu Z. He R. Han J. Huang S.. (2019). Mutagenesis reveals that the OsPPa6 gene is required for enhancing the alkaline tolerance in rice. Front. Plant Sci. 10. doi: 10.3389/fpls.2019.00759 PMC6580931 31244876

[B68] Wei W. H. Hemani G. Haley C. S. (2014). Detecting epistasis in human complex traits. Nat. Rev. Genet. 15 (11), 722–733. doi: 10.1038/nrg3747 25200660

[B69] Wen W. Liu H. Zhou Y. Jin M. Yang N. Li D.. (2016). Combining quantitative genetics approaches with regulatory network analysis to dissect the complex metabolism of the maize kernel. Plant Physiol. 170 (1), 136–146. doi: 10.1104/pp.15.01444 26556794 PMC4704590

[B70] Wurschum T. Maurer H. P. Kraft T. Janssen G. Nilsson C. Reif J. C. (2011). Genome-wide association mapping of agronomic traits in sugar beet. Theor. Appl. Genet. 123 (7), 1121–1131. doi: 10.1007/s00122-011-1653-1 21761161

[B71] Yan S. Zou G. Li S. Wang H. Liu H. Zhai G.. (2011). Seed size is determined by the combinations of the genes controlling different seed characteristics in rice. Theor. Appl. Genet. 123 (7), 1173–1181. doi: 10.1007/s00122-011-1657-x 21805338

[B72] Yang J. Lee S. H. Goddard M. E. Visscher P. M. (2011). GCTA: a tool for genome-wide complex trait analysis. Am. J. Hum. Genet. 88 (1), 76–82. doi: 10.1016/j.ajhg.2010.11.011 21167468 PMC3014363

[B73] Yao M. Wang J. Chen H. Zhai H. Zhang H. (2005). Inheritance and QTL mapping of salt tolerance in rice. Rice Sci. 12 (1), 25–32. Available at: http://www.ricescience.org/CN/Y2005/V12/I1/25.

[B74] Yu S. B. Li J. X. Xu C. G. Tan Y. F. Gao Y. J. Li X. H.. (1997). Importance of epistasis as the genetic basis of heterosis in an elite rice hybrid. Proc. Natl. Acad. Sci. United States America 94 (17), 9226–9231. doi: 10.1073/pnas.94.17.9226 PMC23127 11038567

[B75] Yu Y. Zhang H. Long Y. Shu Y. Zhai J. (2022). Plant public RNA-seq database: a comprehensive online database for expression analysis of ~45 000 plant public RNA-seq libraries. Plant Biotechnol. J. 20 (5), 806–808. doi: 10.1111/pbi.13798 35218297 PMC9055819

[B76] Zhang J.-L. Flowers T. J. Wang S.-M. (2009). Mechanisms of sodium uptake by roots of higher plants. Plant Soil 326 (1-2), 45–60. doi: 10.1007/s11104-009-0076-0

[B77] Zhang Q. Li J. Zhang W. Yan S. Wang R. Zhao J.. (2012). The putative auxin efflux carrier OsPIN3t is involved in the drought stress response and drought tolerance. Plant J. 72 (5), 805–816. doi: 10.1111/j.1365-313X.2012.05121.x 22882529

[B78] Zhang Y. Lin X. Ou X. Hu L. Wang J. Yang C.. (2013). Transcriptome alteration in a rice introgression line with enhanced alkali tolerance. Plant Physiol. Biochem. 68, 111–117. doi: 10.1016/j.plaphy.2013.04.012 23685753

[B79] Zhang H. Liu X. L. Zhang R. X. Yuan H. Y. Wang M. M. Yang H. Y.. (2017). Root damage under alkaline stress is associated with reactive oxygen species accumulation in rice (Oryza sativa l.). Front. Plant Sci. 8. doi: 10.3389/fpls.2017.01580 PMC5596797 28943882

[B80] Zhang J. Singh A. Mueller D. S. Singh A. K. (2015). Genome-wide association and epistasis studies unravel the genetic architecture of sudden death syndrome resistance in soybean. Plant J. 84 (6), 1124–1136. doi: 10.1111/tpj.13069 26561232

[B81] Zhang F. Wang C. Li M. Cui Y. Shi Y. Wu Z.. (2021). The landscape of gene-CDS-haplotype diversity in rice: properties, population organization, footprints of domestication and breeding, and implications for genetic improvement. Mol. Plant 14 (5), 787–804. doi: 10.1016/j.molp.2021.02.003 33578043

[B82] Zhang F. Zeng D. Zhang C. S. Lu J. L. Chen T. J. Xie J. P.. (2019). Genome-wide association analysis of the genetic basis for sheath blight resistance in rice. Rice (N Y) 12 (1), 93. doi: 10.1186/s12284-019-0351-5 31853678 PMC6920286

[B83] Zhang F. Zhai H.-Q. Paterson A. H. Xu J.-L. Gao Y.-M. Zheng T.-Q.. (2011). Dissecting genetic networks underlying complex phenotypes: the theoretical framework. PloS One 6 (1), e14541. doi: 10.1371/journal.pone.0014541 21283795 PMC3024316

[B84] Zhao C. Zhang H. Song C. Zhu J.-K. Shabala S. (2020). Mechanisms of plant responses and adaptation to soil salinity. Innovation 1 (1), 100017. doi: 10.1016/j.xinn.2020.100017 34557705 PMC8454569

[B85] Zhou H. Li J. Liu X. Wei X. He Z. Hu L.. (2021). The divergent roles of the rice bcl-2 associated athanogene (BAG) genes in plant development and environmental responses. Plants (Basel) 10 (10), 2169. doi: 10.3390/plants10102169 34685978 PMC8538510

