# Supplementary material for: Genome-wide association and epistasis studies reveal the genetic basis of saline-alkali tolerance at the germination stage in rice
Source: Front Plant Sci. 2023 May 11;14:1170641. doi: 10.3389/fpls.2023.1170641 (PMC10213895; doi:10.3389/fpls.2023.1170641)
Supplement: Supplementary file 1 [file DataSheet_1.pdf]

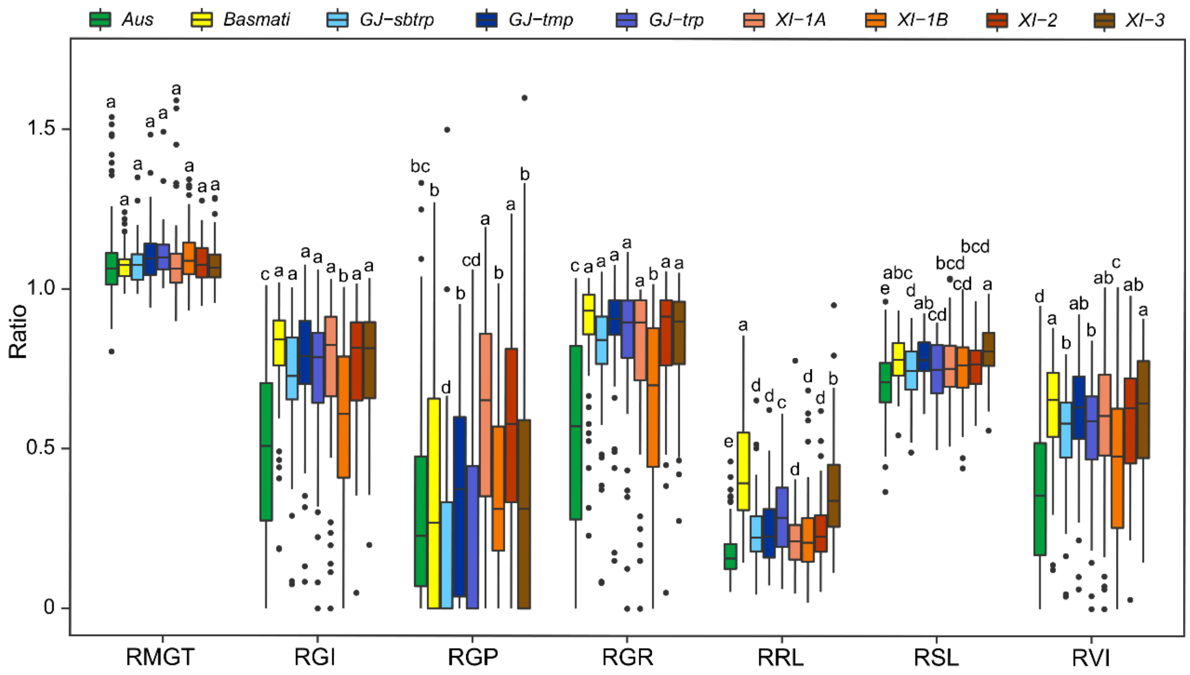

Supplementary Figure 1. Phenotypic distribution of relative saline-alkali damage of measured traits in nine rice subgroups. The population structure of 3KRG and the division of the nine subgroups were reported by Wang et al. (Wang et al., 2018b). Different letters above the boxplot indicate significant differences among subgroups based on Duncan's multiple range post-hoc test ($P < 0.05$).

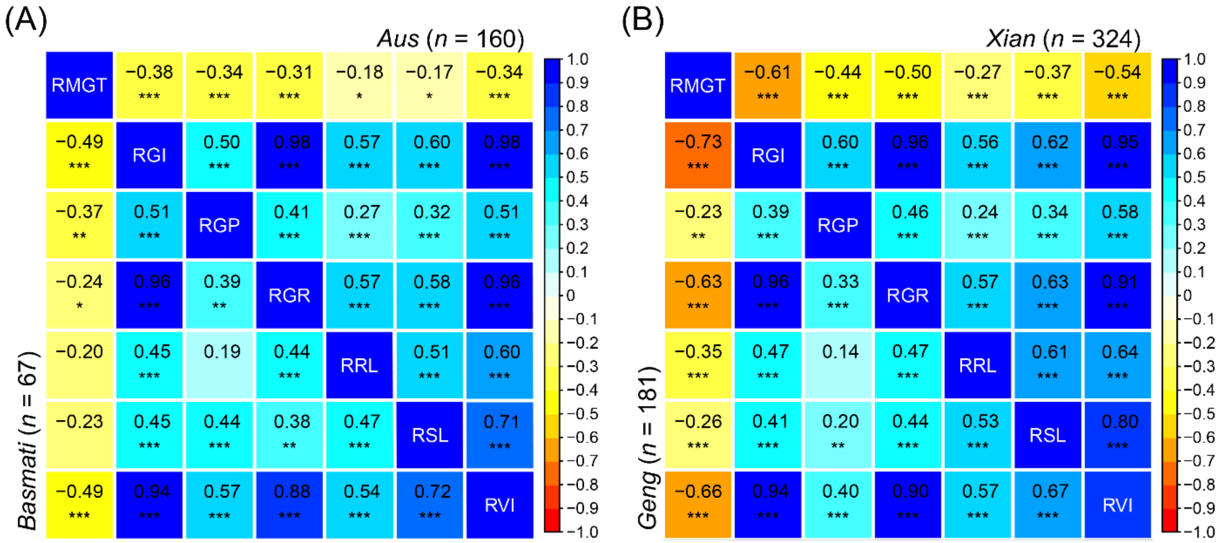

Supplementary Figure 2. Correlations between saline-alkali tolerance-related traits in four subpopulations. (**A**) The upper and lower diagonals show the correlation coefficients in *Aus* and *Basmati*, respectively. (**B**) The upper and lower diagonals show the correlation coefficients in *Xian*/*indica* and *Geng*/*japonica*, respectively. *$P < 0.05$, **$P < 0.01$, and ***$P < 0.001$.

Reference

Abbasi, F., Onodera, H., Toki, S., Tanaka, H., and Komatsu, S. (2004). OsCDPK13, a calcium-dependent protein kinase gene from rice, is induced by cold and gibberellin in rice leaf sheath. *Plant Molecular Biology* 55(4), 541-552. doi: 10.1007/s11103-004-1178-y.

Bhattacharjee, A., Sharma, R., and Jain, M. (2017). Over-Expression of OsHOX24 Confers Enhanced Susceptibility to Abiotic Stresses in Transgenic Rice via Modulating Stress-Responsive Gene Expression. *Front Plant Sci* 8, 628. doi: 10.3389/fpls.2017.00628.

Chapagain, S., Park, Y.C., Kim, J.H., and Jang, C.S. (2018). Oryza sativa salt-induced RING E3 ligase 2 (OsSIRP2) acts as a positive regulator of transketolase in plant response to salinity and osmotic stress. *Planta* 247(4), 925-939. doi: 10.1007/s00425-017-2838-x.

Chen, G., Hu, Q., Luo, L., Yang, T., Zhang, S., Hu, Y., et al. (2015). Rice potassium transporter OsHAK1 is essential for maintaining potassium-mediated growth and functions in salt tolerance over low and high potassium concentration ranges. *Plant Cell Environ* 38(12), 2747-2765. doi: 10.1111/pce.12585.

Duan, J., Zhang, M., Zhang, H., Xiong, H., Liu, P., Ali, J., et al. (2012). OsMIOX, a myo-inositol oxygenase gene, improves drought tolerance through scavenging of reactive oxygen species in rice (Oryza sativa L.). *Plant Sci 196*, 143-151. doi: 10.1016/j.plantsci.2012.08.003.

Duan, Y.B., Li, J., Qin, R.Y., Xu, R.F., Li, H., Yang, Y.C., et al. (2016). Identification of a regulatory element responsible for salt induction of rice OsRAV2 through ex situ and in situ promoter analysis. *Plant Mol Biol* 90(1-2), 49-62. doi: 10.1007/s11103-015-0393-z.

Fanata, W.I., Lee, K.H., Son, B.H., Yoo, J.Y., Harmoko, R., Ko, K.S., et al. (2013). N-glycan maturation is crucial for cytokinin-mediated development and cellulose synthesis in Oryza sativa. *Plant J* 73(6), 966-979. doi: 10.1111/tpj.12087.

Fukuda, A., Nakamura, A., Hara, N., Toki, S., and Tanaka, Y. (2011). Molecular and functional analyses of rice NHX-type Na^+/H^+ antiporter genes. *Planta* 233(1), 175-188. doi: 10.1007/s00425-010-1289-4.

Hauser, F., and Horie, T. (2010). A conserved primary salt tolerance mechanism mediated by HKT transporters: a mechanism for sodium exclusion and maintenance of high K^+/Na^+ratio in leaves during salinity stress. *Plant Cell Environ* 33(4), 552-565. doi: 10.1111/j.1365-3040.2009.02056.x.

Hou, F.-Y., Huang, J., Yu, S.-L., and Zhang, H.-S. (2007). The 6-phosphogluconate Dehydrogenase Genes Are Responsive to Abiotic Stresses in Rice. *Journal of Integrative Plant Biology* 49(5), 655-663. doi: 10.1111/j.1744-7909.2007.00460.x.

Huang, J., Wang, M.-M., Jiang, Y., Bao, Y.-M., Huang, X., Sun, H., et al. (2008). Expression analysis of rice A20/AN1-type zinc finger genes and characterization of ZFP177 that contributes to temperature stress tolerance. *Gene* 420(2), 135-144. doi: 10.1016/j.gene.2008.05.019.

Huang, J., Yang, X., Wang, M.-M., Tang, H.-J., Ding, L.-Y., Shen, Y., et al. (2007). A novel rice C2H2-type zinc finger protein lacking DLN-box/EAR-motif plays a role in salt tolerance. *Biochimica et Biophysica Acta (BBA) - Gene Structure and Expression* 1769(4), 220-227. doi: 10.1016/j.bbaexp.2007.02.006.

Huang, X., Huang, L., Zhao, X., Jia, J., Zhang, G., Zhang, M., et al. (2022). A J-Protein OsDjC46 Interacts with ZFP36 to Participate in ABA-Mediated Antioxidant Defense in Rice. *Antioxidants (Basel)* 11(2), 207. doi: 10.3390/antiox11020207.

Islam, M.O., Kato, H., Shima, S., Tezuka, D., Matsui, H., and Imai, R. (2019). Functional identification of a rice trehalase gene involved in salt stress tolerance. *Gene* 685, 42-49. doi: 10.1016/j.gene.2018.10.071.

Jain, M., Tyagi, A.K., and Khurana, J.P. (2006). Overexpression of putative topoisomerase 6 genes from rice confers stress tolerance in transgenic Arabidopsis plants. *FEBS J* 273(23), 5245-5260. doi: 10.1111/j.1742-4658.2006.05518.x.

Jiang, S.Y., Bhalla, R., Ramamoorthy, R., Luan, H.F., Venkatesh, P.N., Cai, M., et al. (2012). Over-expression of OSRIP18 increases drought and salt tolerance in transgenic rice plants. *Transgenic Res* 21(4), 785-795. doi: 10.1007/s11248-011-9568-9.

Jing, P., Kong, D., Ji, L., Kong, L., Wang, Y., Peng, L., et al. (2021). OsClo5 functions as a transcriptional co-repressor by interacting with OsDi19-5 to negatively affect salt stress tolerance in rice seedlings. *Plant J* 105(3), 800-815. doi: 10.1111/tpj.15074.

Joshi, R., Karan, R., Singla-Pareek, S.L., and Pareek, A. (2016). Ectopic expression of Pokkali phosphoglycerate kinase-2 (OsPGK2-P) improves yield in tobacco plants under salinity stress. *Plant Cell Rep* 35(1), 27-41. doi: 10.1007/s00299-015-1864-z.

Kaur, V., Yadav, S.K., Wankhede, D.P., Pulivendula, P., Kumar, A., and Chinnusamy, V. (2020). Cloning and characterization of a gene encoding MIZ1, a domain of unknown function protein and its role in salt and drought stress in rice. *Protoplasma* 257(2), 475-487. doi: 10.1007/s00709-019-01452-5.

Kumar, M., Choi, J., An, G., and Kim, S.R. (2017). Ectopic Expression of OsSta2 Enhances Salt Stress Tolerance in Rice. *Front Plant Sci* 8, 316. doi: 10.3389/fpls.2017.00316.

Kumar, R., Mustafiz, A., Sahoo, K.K., Sharma, V., Samanta, S., Sopory, S.K., et al. (2012). Functional screening of cDNA library from a salt tolerant rice genotype Pokkali identifies mannose-1-phosphate guanyl transferase gene (OsMPG1) as a key member of salinity stress response. *Plant Mol Biol* 79(6), 555-568. doi: 10.1007/s11103-012-9928-8.

Li, N., Kong, L., Zhou, W., Zhang, X., Wei, S., Ding, X., et al. (2013). Overexpression of Os2H16 enhances resistance to phytopathogens and tolerance to drought stress in rice. *Plant Cell, Tissue and Organ Culture (PCTOC)* 115(3), 429-441. doi: 10.1007/s11240-013-0374-3.

Li, N., Sun, J., Wang, J., Liu, H., Zheng, H., Yang, L., et al. (2017). QTL analysis for alkaline tolerance of rice and verification of a major QTL. *Plant Breeding* 136(6), 881-891. doi: 10.1111/pbr.12539.

Liang, W.-h., Bi, J.-j., Peng, W.-f., Zhang, F., Shi, H.-h., and Li, L. (2010). Cloning and Expression Analysis of a Mitogen-Activated Protein Kinase Gene OsMPK14 in Rice. *Rice Science* 17(4), 269-275. doi: https://doi.org/10.1016/S1672-6308(09)60026-3.

Lin, F., Li, S., Wang, K., Tian, H., Gao, J., Zhao, Q., et al. (2020). A leucine-rich repeat receptor-like kinase, OsSTLK, modulates salt tolerance in rice. *Plant Science : an International Journal of Experimental Plant Biology* 296, 110465. doi: 10.1016/j.plantsci.2020.110465.

Lin, W., Wang, Y., Liu, X., Shang, J.X., and Zhao, L. (2021). OsWAK112, A Wall-Associated Kinase, Negatively Regulates Salt Stress Responses by Inhibiting Ethylene Production. *Front Plant Sci* 12, 751965. doi: 10.3389/fpls.2021.751965.

Liu, C., Mao, B., Ou, S., Wang, W., Liu, L., Wu, Y., et al. (2014). OsbZIP71, a bZIP transcription factor, confers salinity and drought tolerance in rice. *Plant Mol Biol* 84(1-2), 19-36. doi: 10.1007/s11103-013-0115-3.

Liu, C., Wu, Y., and Wang, X. (2012). bZIP transcription factor OsbZIP52/RISBZ5: a potential negative regulator of cold and drought stress response in rice. *Planta* 235(6), 1157-1169. doi: 10.1007/s00425-011-1564-z.

Lou, D., Wang, H., and Yu, D. (2018). The sucrose non-fermenting-1-related protein kinases SAPK1 and SAPK2 function collaboratively as positive regulators of salt stress tolerance in rice. *BMC Plant Biol* 18(1), 203. doi: 10.1186/s12870-018-1408-0.

Lu, W., Deng, M., Guo, F., Wang, M., Zeng, Z., Han, N., et al. (2016). Suppression of OsVPE3 Enhances Salt Tolerance by Attenuating Vacuole Rupture during Programmed Cell Death and Affects Stomata Development in Rice. *Rice (N Y)* 9(1), 65. doi: 10.1186/s12284-016-0138-x.

Luo, C., Guo, C., Wang, W., Wang, L., and Chen, L. (2014). Overexpression of a new stress-repressive gene OsDSR2 encoding a protein with a DUF966 domain increases salt and simulated drought stress sensitivities and reduces ABA sensitivity in rice. *Plant Cell Rep* 33(2), 323-336. doi: 10.1007/s00299-013-1532-0.

Ma, W., Wu, F., Sheng, P., Wang, X., Zhang, Z., Zhou, K., et al. (2017). The LBD12-1 Transcription Factor Suppresses Apical Meristem Size by Repressing Argonaute 10 Expression. *Plant Physiol* 173(1), 801-811. doi: 10.1104/pp.16.01699.

Manimaran, P., Venkata Reddy, S., Moin, M., Raghurami Reddy, M., Yugandhar, P., Mohanraj, S.S., et al. (2017). Activation-tagging in indica rice identifies a novel transcription factor subunit, NF-YC13 associated with salt tolerance. *Sci Rep* 7(1), 9341. doi: 10.1038/s41598-017-10022-9.

Mathan, J., Singh, A., and Ranjan, A. (2021). Sucrose transport in response to drought and salt stress involves ABA-mediated induction of OsSWEET13 and OsSWEET15 in rice. *Physiologia Plantarum* 171(4), 620-637. doi: 10.1111/ppl.13210.

Moon, S.J., Min, M.K., Kim, J.A., Kim, D.Y., Yoon, I.S., Kwon, T.R., et al. (2019). Ectopic Expression of OsDREB1G, a Member of the OsDREB1 Subfamily, Confers Cold Stress Tolerance in Rice. *Front Plant Sci* 10, 297. doi: 10.3389/fpls.2019.00297.

Mukherjee, K., Choudhury, A.R., Gupta, B., Gupta, S., and Sengupta, D.N. (2006). An ABRE-binding factor, OSBZ8, is highly expressed in salt tolerant cultivars than in salt sensitive cultivars of indica rice. *BMC Plant Biol* 6, 18. doi: 10.1186/1471-2229-6-18.

Mukhopadhyay, A., Vij, S., and Tyagi, A.K. (2004). Overexpression of a zinc-finger protein gene from rice confers tolerance to cold, dehydration, and salt stress in transgenic tobacco. *Proceedings of the National Academy of Sciences of the United States of America* 101(16), 6309-6314. doi: 10.1073/pnas.0401572101.

Nakashima, K., Tran, L.S., Van Nguyen, D., Fujita, M., Maruyama, K., Todaka, D., et al. (2007). Functional analysis of a NAC-type transcription factor OsNAC6 involved in abiotic and biotic stress-responsive gene expression in rice. *Plant J* 51(4), 617-630. doi: 10.1111/j.1365-313X.2007.03168.x.

Ni, L., Wang, S., Shen, T., Wang, Q., Chen, C., Xia, J., et al. (2020). Calcium/calmodulin-dependent protein kinase OsDMI3 positively regulates saline-alkaline tolerance in rice roots. *Plant Signal Behav* 15(11), 1813999. doi: 10.1080/15592324.2020.1813999.

Ning, Y., Xie, Q., and Wang, G.L. (2011). OsDIS1-mediated stress response pathway in rice. *Plant Signal Behav* 6(11), 1684-1686. doi: 10.4161/psb.6.11.17916.

Park, H.J., Lee, S.S., You, Y.N., Yoon, D.H., Kim, B.G., Ahn, J.C., et al. (2013). A Rice Immunophilin Gene, OsFKBP16-3, Confers Tolerance to Environmental Stress in Arabidopsis and Rice. *Int J Mol Sci* 14(3), 5899-5919. doi: 10.3390/ijms14035899.

Park, J.J., Yi, J., Yoon, J., Cho, L.H., Ping, J., Jeong, H.J., et al. (2011). OsPUB15, an E3 ubiquitin ligase, functions to reduce cellular oxidative stress during seedling establishment. *Plant J* 65(2), 194-205. doi: 10.1111/j.1365-313X.2010.04416.x.

Peethambaran, P.K., Glenz, R., Honinger, S., Shahinul Islam, S.M., Hummel, S., Harter, K., et al. (2018). Salt-inducible expression of OsJAZ8 improves resilience against salt-stress. *BMC Plant Biol* 18(1), 311. doi: 10.1186/s12870-018-1521-0.

Qiu, T., Zhao, X., Feng, H., Qi, L., Yang, J., Peng, Y.L., et al. (2021). OsNBL3, a mitochondrion-localized pentatricopeptide repeat protein, is involved in splicing nad5 intron 4 and its disruption causes lesion mimic phenotype with enhanced resistance to biotic and abiotic stresses. *Plant Biotechnol J* 19(11), 2277-2290. doi: 10.1111/pbi.13659.

Quinet, M., Ndayiragije, A., Lefevre, I., Lambillotte, B., Dupont-Gillain, C.C., and Lutts, S. (2010). Putrescine differently influences the effect of salt stress on polyamine metabolism and ethylene synthesis in rice cultivars differing in salt resistance. *J Exp Bot* 61(10), 2719-2733. doi: 10.1093/jxb/erq118.

Ray, S., Kapoor, S., and Tyagi, A.K. (2012). Analysis of transcriptional and upstream regulatory sequence activity of two environmental stress-inducible genes, NBS-Str1 and BLEC-Str8, of rice. *Transgenic Res* 21(2), 351-366. doi: 10.1007/s11248-011-9535-5.

Saijo, Y., Kinoshita, N., Ishiyama, K., Hata, S., Kyozuka, J., Hayakawa, T., et al. (2001). A Ca(2+)-dependent protein kinase that endows rice plants with cold- and salt-stress tolerance functions in vascular bundles. *Plant & Cell Physiology* 42(11), 1228-1233. doi: 10.1093/pcp/pce158.

Sakuraba, Y., Piao, W., Lim, J.H., Han, S.H., Kim, Y.S., An, G., et al. (2015). Rice ONAC106 Inhibits Leaf Senescence and Increases Salt Tolerance and Tiller Angle. *Plant Cell Physiol* 56(12), 2325-2339. doi: 10.1093/pcp/pcv144.

Schmidt, R., Schippers, J.H., Mieulet, D., Obata, T., Fernie, A.R., Guiderdoni, E., et al. (2013). MULTIPASS, a rice R2R3-type MYB transcription factor, regulates adaptive growth by integrating multiple hormonal pathways. *Plant J* 76(2), 258-273. doi: 10.1111/tpj.12286.

Seo, J.S., Joo, J., Kim, M.J., Kim, Y.K., Nahm, B.H., Song, S.I., et al. (2011). OsbHLH148, a basic helix-loop-helix protein, interacts with OsJAZ proteins in a jasmonate signaling pathway leading to drought tolerance in rice. *Plant J* 65(6), 907-921. doi: 10.1111/j.1365-313X.2010.04477.x.

Shan, C., Mei, Z., Duan, J., Chen, H., Feng, H., and Cai, W. (2014). OsGA2ox5, a gibberellin metabolism enzyme, is involved in plant growth, the root gravity response and salt stress. *PLoS One* 9(1), e87110. doi: 10.1371/journal.pone.0087110.

Shi, C.C., Feng, C.C., Yang, M.M., Li, J.L., Li, X.X., Zhao, B.C., et al. (2014). Overexpression of the receptor-like protein kinase genes AtRPK1 and OsRPK1 reduces the salt tolerance of Arabidopsis thaliana. *Plant Sci* 217-218, 63-70. doi: 10.1016/j.plantsci.2013.12.002.

Shi, Y., Chang, Y.L., Wu, H.T., Shalmani, A., Liu, W.T., Li, W.Q., et al. (2020a). OsRbohB-mediated ROS production plays a crucial role in drought stress tolerance of rice. *Plant Cell Rep* 39(12), 1767-1784. doi: 10.1007/s00299-020-02603-2.

Shi, Y., Phan, H., Liu, Y., Cao, S., Zhang, Z., Chu, C., et al. (2020b). Glycosyltransferase OsUGT90A1 helps protect the plasma membrane during chilling stress in rice. *J Exp Bot* 71(9), 2723-2739. doi: 10.1093/jxb/eraa025.

Shin, J.-H., Yoshimoto, K., Ohsumi, Y., Jeon, J.-S., and An, G. (2009). OsATG10b, an autophagosome component, is needed for cell survival against oxidative stresses in rice. *Molecules and Cells* 27(1), 67-74. doi: 10.1007/s10059-009-0006-2.

Singh, A.K., Kumar, R., Pareek, A., Sopory, S.K., and Singla-Pareek, S.L. (2012). Overexpression of rice CBS domain containing protein improves salinity, oxidative, and heavy metal tolerance in transgenic tobacco. *Molecular Biotechnology* 52(3), 205-216. doi: 10.1007/s12033-011-9487-2.

Song, T., Shi, Y., Shen, L., Cao, C., Shen, Y., Jing, W., et al. (2021). An endoplasmic reticulum-localized cytochrome b(5) regulates high-affinity K^+transport in response to salt stress in rice. *Proc Natl Acad Sci U S A* 118(50), e2114347118. doi: 10.1073/pnas.2114347118.

Sripinyowanich, S., Chamnanmanoontham, N., Udomchalothorn, T., Maneeprasopsuk, S., Santawee, P., Buaboocha, T., et al. (2013a). Overexpression of a partial fragment of the salt-responsive gene OsNUC1 enhances salt adaptation in transgenic Arabidopsis thaliana and rice (Oryza sativa L.) during salt stress. *Plant Science* 213, 67-78. doi: 10.1016/j.plantsci.2013.08.013.

Sripinyowanich, S., Klomsakul, P., Boonburapong, B., Bangyeekhun, T., Asami, T., Gu, H., et al. (2013b). Exogenous ABA induces salt tolerance in indica rice (Oryza sativa L.): The role of OsP5CS1 and OsP5CR gene expression during salt stress. *Environmental and Experimental Botany* 86, 94-105. doi: 10.1016/j.envexpbot.2010.01.009.

Sun, S.J., Guo, S.Q., Yang, X., Bao, Y.M., Tang, H.J., Sun, H., et al. (2010). Functional analysis of a novel Cys2/His2-type zinc finger protein involved in salt tolerance in rice. *J Exp Bot* 61(10), 2807-2818. doi: 10.1093/jxb/erq120.

Suzuki, K., Yamaji, N., Costa, A., Okuma, E., Kobayashi, N.I., Kashiwagi, T., et al. (2016). OsHKT1;4-mediated Na(+) transport in stems contributes to Na(+) exclusion from leaf blades of rice at the reproductive growth stage upon salt stress. *BMC Plant Biol* 16, 22. doi: 10.1186/s12870-016-0709-4.

Tan, J., Tan, Z., Wu, F., Sheng, P., Heng, Y., Wang, X., et al. (2014). A novel chloroplast-localized pentatricopeptide repeat protein involved in splicing affects chloroplast development and abiotic stress response in rice. *Mol Plant* 7(8), 1329-1349. doi: 10.1093/mp/ssu054.

Tang, Y., Wang, M., Cao, L., Dang, Z., Ruan, N., Wang, Y., et al. (2022). OsUGE3-mediated cell wall polysaccharides accumulation improves biomass production, mechanical strength, and salt tolerance. *Plant Cell Environ* 45(8), 2492-2507. doi: 10.1111/pce.14359.

Thitisaksakul, M., Dong, S., and Beckles, D.M. (2017). How rice Glycogen Synthase Kinase-like 5 (OsGSK5) integrates salinity stress response to source-sink adaptation: A proposed model. *Plant Signal Behav* 12(12), e1403708. doi: 10.1080/15592324.2017.1403708.

Tian, X.-H., Li, X.-P., Zhou, H.-L., Zhang, J.-S., Gong, Z.-Z., and Chen, S.-Y. (2005). OsDREB4 Genes in Rice Encode AP2-Containing Proteins that Bind Specifically to the Dehydration-Responsive Element. *Journal of Integrative Plant Biology* 47(4), 467-476. doi: 10.1111/j.1744-7909.2005.00028.x.

Tian, Y., Zeng, H., Wu, J., Huang, J., Gao, Q., Tang, D., et al. (2022). Screening DHHCs of S-acylated proteins using an OsDHHC cDNA library and bimolecular fluorescence complementation in rice. *The Plant Journal : For Cell and Molecular Biology* 110(6), 1763-1780. doi: 10.1111/tpj.15769.

Wang, H., Zhang, M., Guo, R., Shi, D., Liu, B., Lin, X., et al. (2012). Effects of salt stress on ion balance and nitrogen metabolism of old and young leaves in rice (Oryza sativa L.). *BMC Plant Biology* 12, 194. doi: 10.1186/1471-2229-12-194.

Wang, J., Nan, N., Li, N., Liu, Y., Wang, T.J., Hwang, I., et al. (2020). A DNA Methylation Reader-Chaperone Regulator-Transcription Factor Complex Activates OsHKT1;5 Expression during Salinity Stress. *Plant Cell* 32(11), 3535-3558. doi: 10.1105/tpc.20.00301.

Wang, Y., Li, D., Gao, J., Li, X., Zhang, R., Jin, X., et al. (2017). The 2'-O-methyladenosine nucleoside modification gene OsTRM13 positively regulates salt stress tolerance in rice. *J Exp Bot* 68(7), 1479-1491. doi: 10.1093/jxb/erx061.

Wang, Y., Xiao, Y., Zhang, Y., Chai, C., Wei, G., Wei, X., et al. (2008). Molecular cloning, functional characterization and expression analysis of a novel monosaccharide transporter gene OsMST6 from rice (Oryza sativa L.). *Planta* 228(4), 525-535. doi: 10.1007/s00425-008-0755-8.

Wei, H., Wang, X., He, Y., Xu, H., and Wang, L. (2021). Clock component OsPRR73 positively regulates rice salt tolerance by modulating OsHKT2;1-mediated sodium homeostasis. *EMBO J* 40(3), e105086. doi: 10.15252/embj.2020105086.

Xiang, J., Ran, J., Zou, J., Zhou, X., Liu, A., Zhang, X., et al. (2013). Heat shock factor OsHsfB2b negatively regulates drought and salt tolerance in rice. *Plant Cell Rep* 32(11), 1795-1806. doi: 10.1007/s00299-013-1492-4.

Xiang, Y., Huang, Y., and Xiong, L. (2007). Characterization of stress-responsive CIPK genes in rice for stress tolerance improvement. *Plant Physiol* 144(3), 1416-1428. doi: 10.1104/pp.107.101295.

Xiong, H., Li, J., Liu, P., Duan, J., Zhao, Y., Guo, X., et al. (2014). Overexpression of OsMYB48-1, a novel MYB-related transcription factor, enhances drought and salinity tolerance in rice. *PLoS One* 9(3), e92913. doi: 10.1371/journal.pone.0092913.

Xu, G., Cui, Y., Wang, M., Li, M., Yin, X., and Xia, X. (2014). OsMsr9, a novel putative rice F-box containing protein, confers enhanced salt tolerance in transgenic rice and Arabidopsis. *Molecular Breeding* 34(3), 1055-1064. doi: 10.1007/s11032-014-0096-1.

Xu, N., Chu, Y., Chen, H., Li, X., Wu, Q., Jin, L., et al. (2018). Rice transcription factor OsMADS25 modulates root growth and confers salinity tolerance via the ABA-mediated regulatory pathway and ROS scavenging. *PLoS Genet* 14(10), e1007662. doi: 10.1371/journal.pgen.1007662.

Yang, A., Dai, X., and Zhang, W.H. (2012). A R2R3-type MYB gene, OsMYB2, is involved in salt, cold, and dehydration tolerance in rice. *J Exp Bot* 63(7), 2541-2556. doi: 10.1093/jxb/err431. doi: 10.1093/jxb/err431.

Ye, W., Hu, S., Wu, L., Ge, C., Cui, Y., Chen, P., et al. (2016). White stripe leaf 12 (WSL12), encoding a nucleoside diphosphate kinase 2 (OsNDPK2), regulates chloroplast development and abiotic stress response in rice (Oryza sativa L.). *Mol Breed* 36, 57. doi: 10.1007/s11032-016-0479-6.

Yokotani, N., Ichikawa, T., Kondou, Y., Iwabuchi, M., Matsui, M., Hirochika, H., et al. (2013). Role of the rice transcription factor JAmyb in abiotic stress response. *J Plant Res* 126(1), 131-139. doi: 10.1007/s10265-012-0501-y.

Yokotani, N., Ichikawa, T., Kondou, Y., Maeda, S., Iwabuchi, M., Mori, M., et al. (2009a). Overexpression of a rice gene encoding a small C2 domain protein OsSMCP1 increases tolerance to abiotic and biotic stresses in transgenic Arabidopsis. *Plant Mol Biol* 71(4-5), 391-402. doi: 10.1007/s11103-009-9530-x.

Yokotani, N., Ichikawa, T., Kondou, Y., Matsui, M., Hirochika, H., Iwabuchi, M., et al. (2009b). Tolerance to various environmental stresses conferred by the salt-responsive rice gene ONAC063 in transgenic Arabidopsis. *Planta* 229(5), 1065-1075. doi: 10.1007/s00425-009-0895-5.

Yu, H., Du, Q., Campbell, M., Yu, B., Walia, H., and Zhang, C. (2021). Genome-wide discovery of natural variation in pre-mRNA splicing and prioritising causal alternative splicing to salt stress response in rice. *New Phytol* 230(3), 1273-1287. doi: 10.1111/nph.17189.

Yuchun, R.A.O., Ran, J., Sheng, W., Xianmei, W.U., Hanfei, Y.E., Chenyang, P.A.N., et al. (2021). SPL36 Encodes a Receptor-like Protein Kinase that Regulates Programmed Cell Death and Defense Responses in Rice. *Rice (N Y)* 14(1), 34. doi: 10.1186/s12284-021-00475-y.

Zang, A., Xu, X., Neill, S., and Cai, W. (2010). Overexpression of OsRAN2 in rice and Arabidopsis renders transgenic plants hypersensitive to salinity and osmotic stress. *J Exp Bot* 61(3), 777-789. doi: 10.1093/jxb/erp341.

Zhang, F., Zeng, D., Huang, L., Shi, Y., Chen, T., Zhang, F., et al. (2019). Stress-Activated Protein Kinase OsSAPK9 Regulates Tolerance to Salt Stress and Resistance to Bacterial Blight in Rice. *Rice (N Y)* 12(1), 80. doi: 10.1186/s12284-019-0338-2.

Zhang, J., Sun, Y., Zhou, Z., Zhang, Y., Yang, Y., Zan, X., et al. (2022a). OsSCL30 overexpression reduces the tolerance of rice seedlings to low temperature, drought and salt. *Sci Rep* 12(1), 8385. doi: 10.1038/s41598-022-12438-4.

Zhang, L., Sun, X., Li, Y., Luo, X., Song, S., Chen, Y., et al. (2021). Rice Na(+)-Permeable Transporter OsHAK12 Mediates Shoots Na(+) Exclusion in Response to Salt Stress. *Front Plant Sci* 12, 771746. doi: 10.3389/fpls.2021.771746.

Zhang, M., Zhao, R., Huang, K., Huang, S., Wang, H., Wei, Z., et al. (2022b). The OsWRKY63-OsWRKY76-OsDREB1B module regulates chilling tolerance in rice. *The Plant Journal : For Cell and Molecular Biology* 112(2), 383-398. doi: 10.1111/tpj.15950.

Zhang, X.-H., Rao, X.-L., Shi, H.-T., Li, R.-J., and Lu, Y.-T. (2011). Overexpression of a cytosolic glyceraldehyde-3-phosphate dehydrogenase gene OsGAPC3 confers salt tolerance in rice. *Plant Cell, Tissue and Organ Culture (PCTOC)* 107(1), 1-11. doi: 10.1007/s11240-011-9950-6.

Zhang, Z., Liu, H., Sun, C., Ma, Q., Bu, H., Chong, K., et al. (2018). A C2H2 zinc-finger protein OsZFP213 interacts with OsMAPK3 to enhance salt tolerance in rice. *Journal of Plant Physiology* 229, 100-110. doi: 10.1016/j.jplph.2018.07.003.

Zheng, J., Wang, Y., He, Y., Zhou, J., Li, Y., Liu, Q., et al. (2014). Overexpression of an S-like ribonuclease gene, OsRNS4, confers enhanced tolerance to high salinity and hyposensitivity to phytochrome-mediated light signals in rice. *Plant Science : an International Journal of Experimental Plant Biology* 214, 99-105. doi: 10.1016/j.plantsci.2013.10.003.

Zhu, N., Cheng, S., Liu, X., Du, H., Dai, M., Zhou, D.X., et al. (2015). The R2R3-type MYB gene OsMYB91 has a function in coordinating plant growth and salt stress tolerance in rice. *Plant Sci* 236, 146-156. doi: 10.1016/j.plantsci.2015.03.023.

Zou, J., Liu, C., Liu, A., Zou, D., and Chen, X. (2012). Overexpression of OsHsp17.0 and OsHsp23.7 enhances drought and salt tolerance in rice. *J Plant Physiol* 169(6), 628-635. doi: 10.1016/j.jplph.2011.12.014.
